# Transcription initiation of distant core promoters in a large-sized genome of an insect

**DOI:** 10.1186/s12915-021-01004-5

**Published:** 2021-03-30

**Authors:** Qing Liu, Feng Jiang, Jie Zhang, Xiao Li, Le Kang

**Affiliations:** 1 Beijing Institutes of Life Science, Chinese Academy of Sciences, Beijing, China; 2 Sino-Danish College, University of Chinese Academy of Sciences, Beijing, China; 3 Department of Biology, University of Copenhagen, Copenhagen, Denmark; 4 CAS Center for Excellence in Biotic Interactions, University of Chinese Academy of Sciences, Beijing, China; 5 State Key Laboratory of Integrated Management of Pest Insects and Rodents, Institute of Zoology, Chinese Academy of Sciences, Beijing, 100101 China

**Keywords:** Transcription initiation, Transcriptional start sites, Core promoter, Genome size, Insects

## Abstract

**Background:**

Core promoters have a substantial influence on various steps of transcription, including initiation, elongation, termination, polyadenylation, and finally, translation. The characterization of core promoters is crucial for exploring the regulatory code of transcription initiation. However, the current understanding of insect core promoters is focused on those of Diptera (especially *Drosophila*) species with small genome sizes.

**Results:**

Here, we present an analysis of the transcription start sites (TSSs) in the migratory locust, *Locusta migratoria*, which has a genome size of 6.5 Gb. The genomic differences, including lower precision of transcription initiation and fewer constraints on the distance from transcription factor binding sites or regulatory elements to TSSs, were revealed in locusts compared with *Drosophila* insects. Furthermore, we found a distinct bimodal log distribution of the distances from the start codons to the core promoters of locust genes. We found stricter constraints on the exon length of mRNA leaders and widespread expression activity of the distant core promoters in locusts compared with fruit flies. We further compared core promoters in seven arthropod species across a broad range of genome sizes to reinforce our results on the emergence of distant core promoters in large-sized genomes.

**Conclusions:**

In summary, our results provide novel insights into the effects of genome size expansion on distant transcription initiation.

**Supplementary Information:**

The online version contains supplementary material available at 10.1186/s12915-021-01004-5.

## Background

The core promoter, which is located towards the 5′ region of a gene on the sense strand, is an upstream regulatory region facilitating the transcription initiation of a protein-coding gene. Core promoters contain necessary sequence features to recruit RNA polymerase II to form transcription initiation complexes and initiate the transcription of protein-coding genes [1]. Core promoters play important roles in gene expression regulation with respect to many aspects of transcription, including initiation, elongation, termination, polyadenylation, and finally, translation. The gene expression regulation based on the core promoter can be achieved by diverse mechanisms, including the transcription initiation mode, diversity in the core promoter composition, interactions of the basal transcription machinery with the core promoter, enhancer-promoter specificity, core promoter-preferential activation, enhancer RNAs, Pol II pausing, transcription termination, Pol II recycling, and translation [2]. The identification and characterization of core promoters is very important to understand how transcription occurs and how gene expression is regulated. The accurate annotation of core promoter architecture is largely dependent on the empirical determination of the 5′ end of mRNA transcripts by generating the expression profiles of transcription start sites (TSSs). High-throughput sequencing combined with oligo-capping, which yields millions of 5′ end sequences derived from 5′ capped mRNA transcripts produced by RNA polymerase II, can generate a genome-wide scale map of TSSs and efficiently contribute to the annotation of the core promoter architecture [3]. Transcription initiation occurs at multiple nucleotide positions within a core promoter region [4]. Therefore, core promoters contain not only a single TSS but also an array of closely located initiation sites. Conceptually, the core promoter is entirely different from alternative promoters, which generate alternative isoforms with either distinct 5′-untranslated regions or coding sequences. Multiple TSSs within the same core promoter usually respond in a similar manner to external stimuli and exhibit the same patterns of tissue specificity [5].

The precise annotation of core promoters not only is necessary for understanding the cis-regulatory elements controlling protein-coding gene transcription but also is crucial for genome annotation. Despite the rapid generation of genome sequences of diverse insect taxa, the current official insect gene sets are mostly derived from RNA sequencing (RNA-seq) assemblies. Owing to the inherent technological limitations RNA-seq, the RNA-seq read coverage is strongly biases towards the 3′ landscape of the transcriptome, and the 5′ ends of transcript models are generally inaccurate [6]. The knowledge of transcription initiation and core promoter features in insects lags far behind that in vertebrates as a consequence of the relatively small number of genome-wide TSS studies conducted so far [7–9]. Owing to the absence of a TSS study comparing the insect core promoter architecture beyond Diptera, the current understanding of insect core promoters is largely restricted to the order Diptera [10]. Therefore, in a large number of studies of insect promoters, the region less than 2 kb upstream of the start codon ATG site (2 kb limitation rule) is considered the putative promoter [11–14]. However, the extent to which this 2 kb limitation rule is valid remains an open question, especially in the large-sized genomes of insect species due to the reduced constraints on gene structure size.

The intron position is one of the critical factors in the regulation of transcription initiation [15]. Although a large majority of introns are located within open reading frames, introns in mRNA leaders (5′-UTRs) are common in complex eukaryotes [16]. Introns in mRNA leaders are spliced out before protein translation occurs. Although functions including promoting transcription and nuclear export have been reported for the introns in mRNA leaders [16], their regulatory role is often overlooked. Because intron size and abundance in mRNA leaders have been analyzed in only a few model organisms [15, 16], the effects of introns in mRNA leaders on transcription initiation have been rarely studied within the context of genome size variation.

The migratory locust, *Locusta migratoria*, is a global species representing a model system with remarkable phenotypic plasticity regulated by gene expression [17–19]. Its genome is approximately 6.5 Gb in size, which is at least 30 times larger than the fruit fly genome. The locust genome has undergone a size expansion in intronic and intergenic regions, resulting in a much larger genome and more loosely organized genes than in *Drosophila melanogaster* [20]. These genomic characteristics make the migratory locust a very important organism for analyzing the effects of genome expansion on core promoter features. However, no study has been performed to identify and characterize the TSSs and core promoters at the genome-wide level in locusts so far. The availability of a comprehensive map of the locust core promoters will provide the opportunity to explore the differences in core promoter characteristics between these two insect species separated by 350 million years of evolution [21].

In this study, we identified TSSs at the genome-wide level using 14 oligo-capping libraries derived from nine tissues or organs of the migratory locust. We identified TSS clusters (TSCs) by clustering individual TSSs along the genome into high-density TSS regions and characterized the core promoter features of locusts. We compared the general characteristics of the core promoter features and dynamics between locusts and fruit flies. Furthermore, we unexpectedly detected widespread distant transcription initiation and explored the distinct aspects of distant core promoters in locusts. In addition, we further identified TSCs in seven arthropod species across a broad range of genome sizes, and we revealed specific characteristics of transcription factor (TF) binding sites (TFBSs) of distant transcription initiation in the context of genome size.

## Results

### Identification of transcription start sites and their clusters

**Fig. 1:**
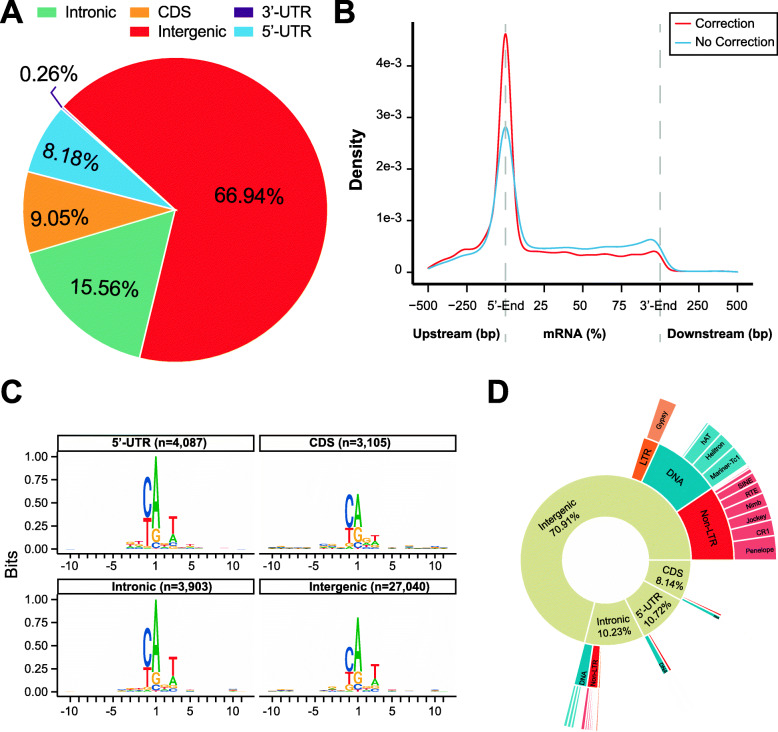
Characteristics of TSSs and TSCs in locusts. **a** Distribution of OTSSs in different genomic regions. **b** Metaprofile of TSCs across the gene bodies of protein-coding genes in the official gene set of locusts. **c** Consensus 25-bp sequences surrounding the dominant TSSs in different genomic regions. The symbol height within the stack indicates the relative frequency of each nucleic acid at that position. The frequency of each nucleotide for each position was represented using the R package Seqlogo. **d** Sunburst charts summarizing the identified TSCs that are derived from TEs

To identify TSSs in the migratory locust, we mapped the oligo-capping sequencing reads from 14 libraries obtained from nine different tissues and organs, including the ovary, testis, wing, thoracic muscle, pronotum, labipalp, brain, fat body, and antenna (Additional file 1: Table S1). All of the oligo-capping libraries were sequenced using an Illumina NovaSeq 6000 System (150-bp paired-end reads). The sequencing of the oligo-capping libraries yielded 1893 million sequencing reads (284 Gb) in total, providing an unprecedented dataset for investigating the 5′ transcriptional start sites of mRNA transcripts in locusts. Only the read pairs that contained both the 5′ oligo-capping and 3′ oligo-capping adapters were mapped to the locust reference genome, with a mean mapping rate of 74.91%. The sequencing read pairs that were properly mapped to the reference genome were used for further analyses. The individual OTSSs were clustered along the genome into TSS clusters. The nucleotide composition of the OTSSs confirmed the absence of systematic G nucleotide addition bias, which is usually observed in the cap analysis gene expression technique (Additional file 1: Fig. S1). The number of OTSSs identified in each library ranged from 290,320 to 1,555,558, with a mean of 615,362 (Additional file 1: Fig. S2). The OTSS number (5,230,229) identified from the combined data of all the libraries was 3.36–18.02 times the number of TSCs identified in any single library (Additional file 1: Fig. S3), suggesting the necessity of investigating more tissues and organs to obtain a more comprehensive TSS landscape in locusts. The OTSSs located within the 1000 bp upstream of the translation start codon formed a broad distribution (Additional file 1: Fig. S4). As expected, the majority of OTSSs (66.94%) were mapped to the intergenic regions (Fig. 1a), indicating that widespread transcription is initiated from noncoding regions in the locust genome.

We identified TSCs, which are high-density regions of TSSs, by clustering individual OTSSs along the genome into TSS clusters. To avoid false TSCs, the TSCs with sequencing reads showing fewer than 3 read tags of TSCs per million (TPM) were not used in further analyses because they probably come from truncated transcripts and cryptic transcripts due to the inherent nature of the basic transcriptional machinery [22]. The number of TSCs identified in each library ranged from 22,858 to 47,615, with a mean of 36,229. The combined data from all libraries yielded 72,280 TSCs, which were used as the initial set of TSCs. In the initial set of TSCs, we observed a large proportion (56.39%) of 1-bp-wide TSCs in the intergenic regions (Additional file 1: Fig. S5).

To discover motifs within TSCs, we generated a consensus sequence logo of 25 bp surrounding the dominant TSSs in each TSC. The composition of [− 1, + 1] initiator dinucleotides revealed a severe overrepresentation of pyrimidine–purine (PyPu) dinucleotides in the non-1-bp-wide TSCs located in the 5′-UTRs, coding DNA sequences (CDSs), and intronic and intergenic regions but not in those located in the 3′-UTRs (Additional file 1: Fig. S6). However, PyPu dinucleotide initiators were absent in all of the 1-bp-wide TSCs except for the 1-bp-wide TSCs located in 5′-UTRs. These results imply that a considerable portion of the 1-bp-wide TSCs were derived from false-positive signals due to the experimental limitation of the oligo-capping method. Thus, we applied additional correction steps to remove putative false TSCs. A large proportion of the identified TSCs showed significant enrichment of the TGAG motif and its 1-bp-substitution variants upstream of the dominant TSS sites of TSCs (Additional file 1: Fig. S7 and Tables S2-S7). These results suggested that these 1-bp-wide TSCs were likely false TSCs derived from mis-hybridization of the 5′ oligo-capping adapters and internal RNA molecules (Additional file 1: Fig. S8). Therefore, the TSCs showing significant enrichment (observed number greater than 20 and a *q*-value of less than 1e−10) of the TGAG motif and its 1-bp-substitution variants were filtered for further analysis. The insert fragments generated in sequencing library construction showed a unimodal distribution with a peak at approximately 300–400 bp. Therefore, the internal false signals were generated from possible truncated mRNAs, and the insert fragment size in the 3′ ends did not obey the insert fragment limits along the mRNA transcripts (Additional file 1: Fig. S9). As expected, the 3′ end distribution (median = 352.3 bp; first quartile to third quartile, 229.1–557.3 bp) of the mean insert fragments inferred by determining the start sites of paired R2 (reverse) reads was consistent with the insert fragment distribution observed in sequencing library construction. The inferred 3′ end distribution of the insert fragments was unimodal and asymmetric, with a long tail to the right (Additional file 1: Fig. S10). Therefore, the TSCs showing deviation of the distance from the 3′ ends of the insert fragment to the start sites of the mRNAs were considered to come from possible truncated mRNAs and were therefore removed from further analysis above the threshold of the 90% quantile (Additional file 1: Fig. S11). It is worth noting that a total of 8247 (2252 in 1-bp-wide TSCs and 5995 in non-1-bp-wide TSCs) intergenic TSCs could be assigned to the mRNAs of protein-coding genes by paired R2 read linking. This result suggested that the start sites of mRNAs in the official gene set do not represent authentic TSS sites because of the technological inability to achieve sufficient 5′-UTR coverage using standard Illumina RNA-seq. Overall, we identified 38,136 TSCs in the final set after removing the false TSCs derived from adapter mis-hybridization and internal truncated sites. The width of most of the identified TSCs (median = 38 bp and 90% quantile = 133 bp in non-1-bp-wide TSCs) in the final set was less than 150 bp, which is consistent with that of *Drosophila* TSCs (median = 36 bp and 90% quantile = 152 bp in non-1-bp-wide TSCs) identified via the RAMPAGE method [23]. The performance assessment for TSC identification was judged on the basis of the distribution of the identified TSCs over gene bodies. Compared with that of the TSCs in the initial set, the higher enrichment of TSCs in the 5′ ends of protein-coding genes in the final set indicated that the application of the correction steps greatly improved the ability to distinguish between authentic TSCs and false TSCs (Fig. 1b). The composition of [− 1, + 1] initiator dinucleotides showed that the PyPu dinucleotide initiators are preferentially used as TSSs in the different genomic regions (Fig. 1c). Thus, these 38,136 TSCs in the final set are reliable TSCs in locusts.

Compared with distance-based promoter identification, promoter identification involving the paired-read-based assignment rule allows the more accurate assignment of core promoters to existing gene models and provides direct evidence for characterizing the transcription start site landscape of protein-coding genes. For each TSC, if an insert fragment with its 5′ end in the TSCs and its 3′ end in an annotated exon of a protein-coding gene was identified, the TSC was functionally linked to the gene. The 38,136 reliable TSCs in the final set were linked to annotated protein-coding genes based on gene structure information using the paired-read-based assignment rule. We thus assigned 48.0% (18,305 in 38,136) of the reliable TSCs to annotated protein-coding genes, and the remaining 52.0% represented potential initiation sites of unannotated nonprotein-coding genes. The comparison of biological replicates of the tissue and organ data confirmed the ability to quantify promoter expression using the oligo-capping method with excellent quantification reproducibility (Additional file 1: Fig. S12, *P*s < 2.2e−16, Pearson’s *R* = 0.99; Pearson correlation coefficients were obtained using the TPM values of all of the detected TSCs). To identify the genic TSCs for which promoter activities are provided by transposable elements (TEs), we searched for TE-containing TSCs that drive the expression of annotated protein-coding genes. We identified a considerable proportion (14.64%, 2779 of 18,305) of reliable TSCs derived from 36 multiple families of TEs that drive the expression of 2190 annotated protein-coding genes. This observation was more prevalent among TSCs located in intergenic regions. Although all three major classes of locust TEs were present (non-LTR, LTR and DNA), the percentage of genic TSCs whose expression was driven by TEs was not directly proportional to the composition of TEs in the locust genome (Fig. 1d). For example, members of the RTE/BovB subfamily (constituting 244 Mb [4.05%] of the locust genome), which is the most prevalent TE subfamily in the locust genome [20], contributed to only 0.07% (2 in 2679) of the genic TSCs.

### Characteristics of locust core promoters

**Fig. 2:**
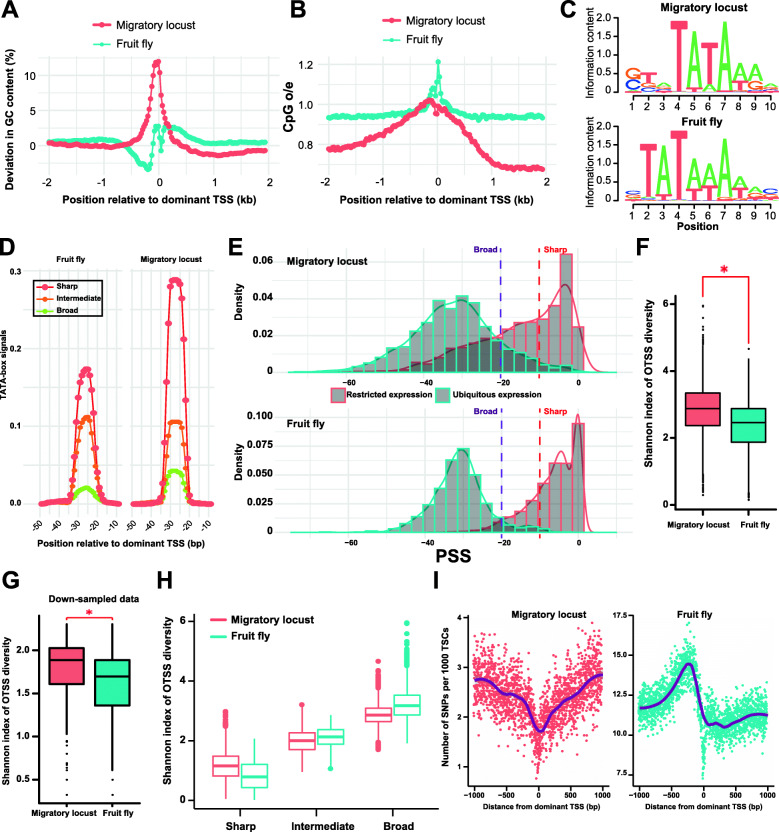
Characterization of core promoters in locusts. **a** Patterns of the GC content in the 2-kb flanking region of transcription start sites. The deviation of the GC content in sliding windows was determined using the GC content normalized to the mean GC content. **b** CpG occurrence in the 2-kb flanking region of transcription start sites. Normalized CpG contents (CpG observed/expected, CpG oe) and GC contents were computed in a 50-bp sliding window across 4-kb regions centered on the dominant TSSs. **c** De novo motif discovery in the 20 to 40 bp region upstream of the dominant OTSSs of core promoters. **d** TATA-box signals in the upstream regions of PyPu dinucleotides in the core promoters with different promoter shapes. **e** Density distribution of the PSS values of promoters with ubiquitous and restricted TSC expression. **f** Shannon index of OTSS diversity in genic core promoters. **g** Shannon index of OTSS diversity in genic core promoters in the down-sampled data. **h** Shannon diversity index of OTSSs in the core promoters with different promoter shapes. **i** Density of SNPs flanking the transcription start sites. The TSCs flanked by repetitive elements were not included in this comparison. The red asterisk indicates *P* < 2.2e−16 according to the Wilcoxon rank-sum test

We obtained 22,820 genic TSCs in *Drosophila melanogaster* (Additional file 1: Fig. S13) and used them to compare core promoter characteristics between locusts and fruit flies [7, 23]. Similar initiator (PyPu dinucleotide; notably, because no mutational analysis was performed, the identified PyPu dinucleotide was considered the overrepresented motif) elements were detected in the genic TSCs of locusts and fruit flies (Additional file 1: Fig. S14A). However, the analysis of the nucleotide composition flanking the PyPu dinucleotide of core promoters in locusts revealed a preference for 2-bp downstream T/A usage, which was not observed in fruit flies. By examining the AT contents of the 2-kb flanking regions of core promoters, we found two striking distinct patterns in the nucleotide composition of locusts and fruit flies. Unlike in fruit flies, enrichment of GC nucleotides in the 500-bp flanking regions of the core promoters of locusts was observed, emphasizing the preferential location of the GC-rich regions of locust core promoters (Fig. 2a). Although there is evidence of DNA methylation in gene bodies and repeat regions in locusts, the status of promoter methylation has not been explored because of the unavailability of promoter data [20, 24]. The relative depletion of CpG dinucleotides is negatively correlated with DNA methylation. Thus, we performed a normalized CpG content analysis to determine whether the increases in GC content were associated with the existence of DNA methylation. The fruit fly exhibited a unimodal (centered on approximately 0.93) normalized CpG content (CpG o/e, CpG observed/expected ratio) distribution (a signal of devoid of DNA methylation), and the CpG o/e values consistently remained at approximately 0.93 in the 2-kb flanking region of PyPu dinucleotide (Additional file 1: Fig. S15). However, the locust exhibited a broad distribution of CpG o/e values (Additional file 1: Fig. S16), and the 2-kb flanking regions of the locust core promoters exhibited signatures of gradual CpG restoration (Fig. 2b) as the distance to the dominant OTSS decreased [25]. Thus, the CpG occurrence peaks (approaching the right side of the bimodal CpG o/e value distribution) at the center of the locust core promoters are suggestive of the absence of DNA methylation in the core promoters of locusts despite the enrichment of GC nucleotides in the 500-bp flanking regions of core promoters.

To identify the well-accepted *Drosophila* core promoter elements, the consensus sequences (Additional file 1: Table S8) of the TATA-box, initiator (Inr), polypyrimidine initiator (TCT), motif ten element (MTE), and downstream core promoter element (DPE) were obtained from a recent review [26]. The consensus sequences were used in pattern matching of the putative *Drosophila* core promoter elements while allowing one mismatch. There was no obvious difference in the percentage of the *Drosophila* core promoter elements between the locust and fruit fly core promoters (Additional file 1: Fig. S14B). We extracted the random genomic sequences of which numbers are equal to the numbers of the genic TSCs identified in locusts (*N* = 18,305) and fruit flies (*N* = 22,820), respectively. The percentages of PyPu dinucleotide in the locust (85.38% in the genic TSCs and 6.87% in the random sequences) and fruit fly (86.49% in the genic TSCs and 23.75% in the random sequences) core promoters are statistically different (*Ps* < 2.2e−16, chi-squared tests) from those in the random sequences. The statistical differences (*Ps* < 2.2e−16, chi-squared tests) remain unchanged for the TATA-box elements in both locusts (18.19% in the genic TSCs and 10.45% in the random sequences) and fruit flies (17.61% in the genic TSCs and 10.47% in the random sequences).

Like in fruit flies, we observed an increase in AT contents in the 20 to 40 bp regions upstream of the PyPu dinucleotide in locusts; these are typical regions in which TATA-box elements are located (Additional file 1: Fig. S17). We performed a de novo motif discovery analysis to identify the potential enriched motifs in the 20 to 40 bp regions upstream of the PyPu dinucleotide in both species. Two different TATA-box motif variants were present in the 20 to 40 bp regions upstream of the PyPu dinucleotide in both species. In fruit flies, the TATA-box motif variant identified by our de novo motif discovery analysis was identical to the TATA-box motif (matrix profile POL012.1) deposited in the JASPAR database [27]. Although the four core nucleotides (TATA) were present in both species, the distinct hallmark of the TATA-box motif variant in locusts was a G/C preference in the 3 bp upstream region of the TATA core nucleotides (Fig. 2c), consistent with the increases in GC nucleotides in locust core promoters. The GC contents in the 3 bp region upstream of the four core nucleotides in locusts and in fruit flies are 92.3% and 53.5%, respectively.

### Imprecise transcription initiation and symmetrical pattern of the SNP density of locust core promoters

Transcription can be initiated at precise genomic regions or dispersed genomic regions, a distinction referred to as promoter shape. Distinct promoter classes are defined based on the shape of the TSS distribution: sharp core promoters or broad core promoters [3, 28]. The sharp and broad structures of core promoters are largely conserved across species and are likely to be associated with different functional motifs, emphasizing distinct functional roles between different shapes [9, 29]. The Promoter Shape Score (PSS), a metric for describing promoter shape, was determined to characterize the shape of the locust core promoters. We classified the core promoters based on PSS values and examined the association between the promoter shape and TATA-box signal. On the basis of a previous study [30], the core promoters were divided into three categories: sharp (PSS > − 10) core promoters, intermediate (PSS ≤ − 10 and PSS > − 20) core promoters, and broad (PSS ≤ − 20) core promoters. Both locusts and fruit flies showed a broad PSS value distribution, suggesting that the transcription can be initiated from precise genomic regions to dispersed genomic regions in insects. We observed an obvious tendency for TATA-box signals to appear in upstream regions of PyPu dinucleotides in sharp core promoters in both locusts and fruit flies. However, the sharp core promoters of locusts showed stronger TATA-box signals than those of fruit flies (Fig. 2d), despite the lower overall TA content in the core promoters of locusts. The broad core promoters driving the transcription of ubiquitously expressed genes were TATA-less promoters in both species [31, 32]. To explore the roles of promoter shape in reflecting TSC expression specificity, we used *τ* to measure the expression specificity. *τ* varies from 0 to 1, where 0 indicates ubiquitous expression and 1 indicates restricted expression. Contrary to protein-coding genes with a bimodal distribution of *τ* scores [33], the *τ* scores in locusts were skewed towards classifying many core promoters as showing restricted expression (Additional file 1: Fig. S18) [34]. We performed Gene Ontology (GO) enrichment analysis to classify the core promoters with ubiquitous and restricted expression according to the functional annotation of linked protein-coding genes. The sets of ubiquitously expressed core promoters were predominantly enriched for GO categories associated with the general/basic functions such as ncRNA processing, translation, and regulation of RNA metabolic processes. Conversely, the core promoters with restricted expression patterns were enriched for specific biological processes related to synaptic transmission, neuron fate specification, regulating TF activities and signaling pathways, and response to light intensity (Additional file 1: Fig. S19). Like in fruit flies, the genic TSCs with ubiquitous expression patterns tended to form broad core promoters in locusts (mean PSS = − 33.62 in locusts and mean PSS = − 30.91 in fruit flies, Fig. 2e). The genic TSCs with restricted expression patterns tended to form sharp core promoters in fruit flies, whereas the genic TSCs with restricted expression patterns exhibited a broader distribution in terms of promoter width in locusts (mean PSS = − 15.87 in locusts and mean PSS = − 6.48 in fruit flies, *P* < 2.2e−16, Wilcoxon rank-sum test). Therefore, both sharp and broad core promoters in locusts can drive the transcription of protein-coding genes with restricted expression.

In both restricted and ubiquitously expressed TSCs, the locusts showed a greater proportion of PSS values towards the left tail (Fig. 2e) than fruit flies. Therefore, we asked whether the imprecision of transcription initiation in locusts is higher than that in fruit flies. Because imprecise transcription initiation in a genic TSC results in an increased number of OTSSs (OTSS diversity), we compared the number of OTSSs of genic TSCs between locusts and fruit flies. We found that the mean number of OTSSs of genic TSCs in locusts was significantly higher than that in fruit flies (*P* < 0.05, Wilcoxon rank-sum test). Furthermore, we assessed the OTSS diversity of genic TSCs using the Shannon index (*H*), which is a diversity index that takes into account not only the OTSS number but also the evenness of the relative usage of different OTSSs [35]. In general, the *H* values of locusts were significantly higher than those of fruit flies (Fig. 2f, mean *H* values = 2.84 in locusts and mean *H* values = 2.35 in fruit flies, *P* < 2.2e−16, Wilcoxon rank-sum test), suggesting increased OTSS diversity of genic TSCs in locusts. To exclude the potential influences of the different TSS profiling methods applied and unequal sequencing depths in the locust and fruit fly data, we performed down-sampling analyses to examine the robustness of the above results. The *P* value of the Wilcoxon rank-sum test remained significant in the down-sampled data (Fig. 2g, mean *H* values = 1.80 in locusts and 1.58 in fruit flies, *P* < 2.2e−16, Wilcoxon rank-sum test). To describe the mean relationship between TSC expression and OTSS diversity using a partitioning method, we grouped the genic TSCs into 10 bins based on their expression quantile ranges. The binscatter plot shows that the TSC expression in both locusts and fruit flies is significantly positively (Additional file 1: Fig. S20, Pearson’s *R* = 0.75 in locusts and 0.51 in fruit flies; *P*s < 2.2e-16) correlated with OTSS diversity, suggesting that increases in TSC expression are generally achieved by the activation of transcription initiation from expanding OTSSs (increasing OTSS diversity within individual genic TSCs) in insects. When the genic TSCs were grouped into three categories based on PSS values, we found that the *H* values of the nonsharp core promoters in locusts were significantly higher than those in fruit flies (Fig. 2h, *P* < 2.2e−16 in the broad core promoters and *P* < 2.2e−16 in the intermediate core promoters, Wilcoxon rank-sum tests). However, a similar observation was not made for the sharp core promoters. Overall, the increased OTSS diversity of genic TSCs indicates a lower precision of transcription initiation of the broad and intermediate core promoters in locusts compared with fruit flies.

Genomic regions flanking TSSs are enriched in functionally important regulatory elements, which show depletion of single-nucleotide polymorphisms (SNPs) due to evolutionary conservation [36]. To determine the sequence variability of the genomic regions flanking TSSs, we extracted 1-kb fragments centered on TSSs and computed the position-specific density of SNPs using the resequencing data of locusts and fruit flies [37, 38]. We found two distinctive patterns of SNP density in the vicinity of dominant OTSSs in the two insect species (Fig. 2i); symmetrical and asymmetrical patterns of SNP density were observed in locusts and in fruit flies, respectively. The steep decline in the SNP density at approximately 250 bp upstream of dominant OTSSs suggests immediate constraints imposed by the presence of TFBSs or regulatory elements. However, the gradual decrease in SNP density from 1 kb upstream to the center of dominant OTSSs in locusts indicated fewer constraints on the distance from TFBSs or regulatory elements to TSSs in locusts than in fruit flies.

### Alternative usage of core promoters of protein-coding genes in locusts

Based on the number of assigned core promoters, the protein-coding genes in locusts are divided into two categories: single-core-promoter genes and multicore-promoter genes. The multicore-promoter genes with two or more core promoters included 38.90% of the assigned protein-coding genes in locusts. The proportion of multicore-promoter genes in locusts was similar to that in fruit flies (38.71%). Compared with the fly genome, in which 37.78% of the genome consists of intergenic regions, the locust genome is greatly expanded, and a total of 73.67% of the locust genome consists of intergenic regions. The average length of the intergenic sequences in locusts was much longer than that in the fruit fly genome (217.63 kb in locusts and 4.42 kb in fruit flies). Therefore, despite the remarkable discrepancy in the sizes of intergenic regions between the species, the proportions of multicore-promoter genes are similar between locusts and fruit flies.

For multicore-promoter genes, the distributions of OTSSs between core promoters differed considerably in different tissues of locusts. The alternative usage of core promoters is also referred to as core-promoter shifting, which is used to quantify OTSS distribution dynamics among core promoters. To determine the prevalence of core-promoter shifting, the degree of shift (*D*_*s*_ value) was determined by calculating changes in the OTSS distribution between core promoters for each multicore-promoter gene. The distribution of *D*_*s*_ values approximately followed a normal distribution (Additional file 1: Fig. S21), implying the dynamic usage of core promoters. We found that 31.09% of multicore-promoter genes have undergone a significant shift in core-promoter usage in at least one tissue or organ (*D*_*s*_ values < − 1 or *D*_*s*_ values > 1, *P* < 0.05 and FDR < 0.1, chi-squared tests, Additional file 1: Fig. S22). This suggests pervasive variability of the 5′-UTR sequences of protein-coding genes in locusts, with implications for translation start site selection in a tissue-specific manner.

### Adjacent and distant core promoters in locusts and flies

**Fig. 3:**
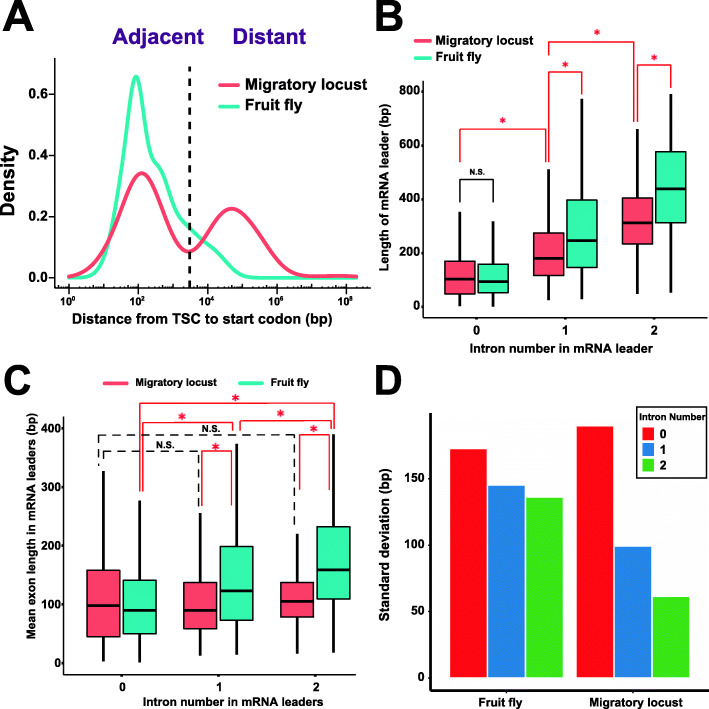
Distant transcription initiation in locusts and fruit flies. **a** The density distribution of distances from the annotated start codon of protein-coding genes to its farthest upstream core promoters. **b** Boxplot showing the length difference of the mRNA leaders (5′-UTRs) with different intron numbers. **c** Boxplot showing the exon length difference in the mRNA leaders with different intron numbers. The red asterisk indicates *P* < 2.2e−16 according to the Wilcoxon rank-sum test. N.S., not significant. **d** Standard deviation of exon lengths in the mRNA leaders with different intron numbers. The red asterisk indicates *P* < 2.2e−16 according to the Wilcoxon rank-sum test. N.S., not significant

The density distribution of the distances from the annotated start codons to the farthest upstream genic core promoters showed a distinctive bimodal log distribution with a valley at 3 kb in locusts (Fig. 3a). However, only a unimodal distribution with a peak at approximately 100 bp was found in fruit flies. Thus, the genome size expansion resulted in a looseness of upstream regulatory elements in locusts. This observation held when all of the core promoters of each protein-coding gene were included (Additional file 1: Fig. S23). Therefore, the core promoters in these two species were classified into the adjacent and distant core promoters using a threshold of 3 kb. Compared with fruit flies (15.48%), locusts exhibited more than triple the number of distant core promoters (45.02%, *P* < 2.2e−16, chi-squared test). Thus, a considerable portion of protein-coding genes contain introns located between the coding and 5′-untranslated first exons, considering that the mean length of mRNA leaders is much shorter than 3 kb.

For the protein-coding genes with annotated mRNA leaders, the intron number in mRNA leaders was less than 3 in both locusts (99.98%) and fruit flies (99.16%), indicating the possibility of strong selection constraints against presenting many introns in mRNA leaders. In mRNA leaders, the intron lengths in locusts (median length = 14,302 bp, 25% quantile = 1957 bp, and 75% quantile = 34,602 bp for the mRNA leaders that have one intron [MLOI]; median length = 17,627 bp, 25% quantile = 8170 bp, and 75% quantile = 46,448 bp for the mRNA leaders that have two introns [MLTI]) were significantly longer (*P*s < 2.2e−16, Wilcoxon rank-sum tests) than those in fruit flies (median length = 929 bp, 25% quantile = 173 bp, and 75% quantile = 3105 bp for MLOI; median length = 3068 bp, 25% quantile = 598 bp, and 75% quantile = 9430 bp for MLTI; Additional file 1: Fig. S24). Furthermore, in both locusts (*P* = 1.303e−06, Wilcoxon rank-sum test) and fruit flies (*P* < 2.2e−16, Wilcoxon rank-sum test), the mean intron length in MLTI was significantly longer than that in MLOI. The median length of the mRNA leaders in locusts was 133 bp (25% quantile = 70 bp and 75% quantile = 230 bp), which was similar (*P* = 0.9652, Wilcoxon rank-sum test) to that in fruit flies (median length = 117 bp, 25% quantile = 67 bp and 75% quantile = 263 bp). Significant increases (*P*s < 2.2e−16, Wilcoxon rank-sum tests) in the length of mRNA leaders were accompanied by increases in intron numbers in both locusts and fruit flies (Fig. 3b). Furthermore, the mRNA leader lengths in locusts were significantly shorter (*P*s < 2.2e−16, Wilcoxon rank-sum tests) than those in fruit flies in both MLOI and MLTI. However, no significant length difference in mRNA leaders between locusts and fruit flies was observed in the mRNA leaders without introns (MLIPs). With respect to the exon-level comparison of mRNA leaders, it was observed only in fruit flies that significant increases in exon length (*P*s < 2.2e−16, Wilcoxon rank-sum tests) were accompanied by increases in intron numbers (Fig. 3c). The exon lengths of both MLOI and MLTI in locusts were significantly shorter (*P*s < 2.2e−16, Wilcoxon rank-sum tests) than those in fruit flies. In addition, a comparison of the standard deviation showed that the exon lengths (mean = 118.5 bp) of MLTI presented less variance than those of either MLIP or MLOI in locusts (Fig. 3d).

**Fig. 4:**
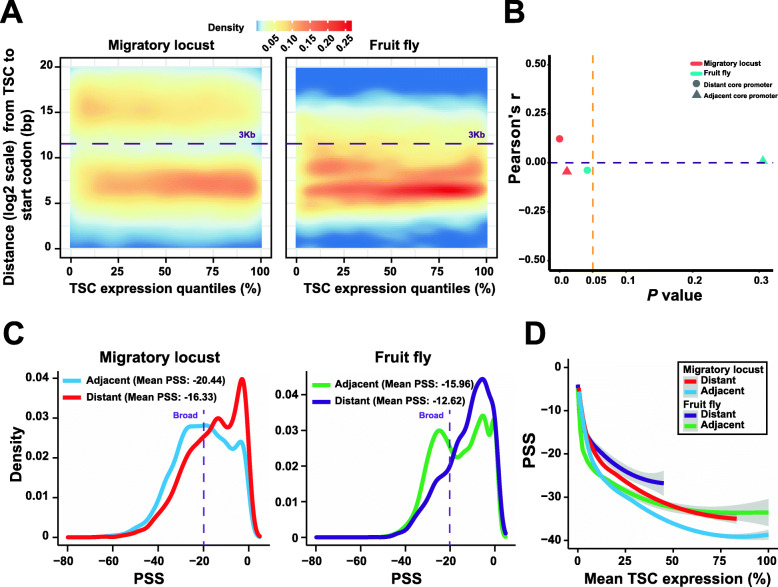
Comparison of adjacent and distant core promoter genes between locusts and fruit flies. **a** Scatter plot of the TSC expression quantiles and the distances from the annotated start codons of protein-coding genes to the core promoters upstream. The density of points is shown using the smooth Scatter kernel-based density function in R. **b** Correlation between the distances from the annotated start codons to the upstream core promoters and TSC expression (TPM). **c** Density distribution of the PSS values of adjacent and distant core promoters. **d** Relationships between genic TSC expression and the shape dynamics of core promoters. All of the core promoters were sorted by the TPM of each genic TSC and were assigned to expression quantiles for each species. For all core promoters, we used a 200-core-promoter window size with a moving step size of 40 core promoters. The data represent the mean PSS values and mean TSC expression, which are normalized on the basis of the maximum TPM value of each category. The smooth lines were plotted with stat_smooth within the R environment using the ggplot2 package

Both the single-core promoters (34.23% in locusts and 4.70% in fruit flies are distant core promoters, *P* < 2.2e−16, chi-squared test) and multicore promoters (68.24% in locusts and 37.90% in fruit flies are distant core promoters, *P* < 2.2e−16, chi-squared test) genes of locusts exhibited a significantly greater number of distant core promoters than those of fruit flies. Furthermore, the multicore-promoter genes presented a significantly greater number of distant core promoters than the single-core-promoter genes did in both locusts and fruit flies (*P*s < 2.2e−16, chi-squared tests). One distinctive difference between the two types of core promoters in the two species was their transcriptional abundance. As expected, the majority of the adjacent core promoters in fruit flies showed higher transcriptional abundance than the distant core promoters. The transcriptional activities of distant core promoters were significantly weaker than those of adjacent core promoters in fruit flies. However, the scatter plot of TSC expression quantiles indicated that a substantial portion of the distant core promoters in locusts showed high transcriptional activity (Fig. 4a). Furthermore, we observed a weak but significant positive correlation (Pearson’s *R* = 0.12 and *P* = 5.192e−15) between the distances from the annotated start codons to the upstream core promoters and TSC expression (TPM) levels in distant core promoters in locusts. However, no similar positive correlation was observed for either the adjacent core promoters in locusts or the adjacent/distant core promoters in fruit flies (Fig. 4b). Broad core promoters were detected in the adjacent core promoters of both locusts and fruit flies (Fig. 4c). In fact, a depletion of distant broad core promoters was observed in the broad core promoters of fruit flies. However, broad core promoters were found in a substantial proportion of distant core promoters in locusts.

To determine whether the overrepresentation of distant broad core promoters in locusts reflects TSC expression specificity, we used *τ* to measure expression specificity. No significant differences in the TSC expression specificity of distant core promoters were detected between locusts and fruit flies (*P*s > 0.05, Wilcoxon rank-sum test), suggesting that both ubiquitous and restricted expression patterns are present in the distant core promoters of locusts and fruit flies. Considering all of these results together, the detection of broad core promoters with restricted expression patterns in locusts reflects only distinct fundamental aspects of gene regulation in locusts and fruit flies, suggesting the lower precision of transcription initiation of locust genic TSCs with restricted expression patterns.

Considering TSC expression in relation to the shape dynamics of core promoters, we performed a sliding window analysis using PSS values and TSC transcriptional abundances to determine the relationship between the core promoter shape and TSC expression. By plotting the PSS values and the expression quantiles of each sliding window, we found that the increases in TSC expression were generally accompanied by decreases in PSS values (Fig. 4d). Enhanced TSC transcriptional abundance gradually results in a broader range of core promoters in both locusts and fruit flies. Therefore, increases in TSC expression are generally achieved by the activation of transcription initiation from expanding OTSSs in insects rather than increased TSC expression of sharp promoters within a few OTSSs, demonstrating a positive correlation between gene expression and PSS values. Compared with distant core promoters, adjacent core promoters showed higher expression activity of genic TSCs in locusts and fruit flies. In addition, similar to fruit flies, the spatial distribution of OTSS signals varied considerably among adjacent core promoters in locusts, spanning a range of promoter shapes from sharp to broad. However, the line termini of the distant core promoters of locusts approached towards those of adjacent core promoters. Thus, unlike fruit flies, the distant core promoters in locusts showed higher expression activities of genic TSCs and became broader with respect to promoter width.

### Distant core promoter emergence in the context of genome size evolution in insects

**Fig. 5:**
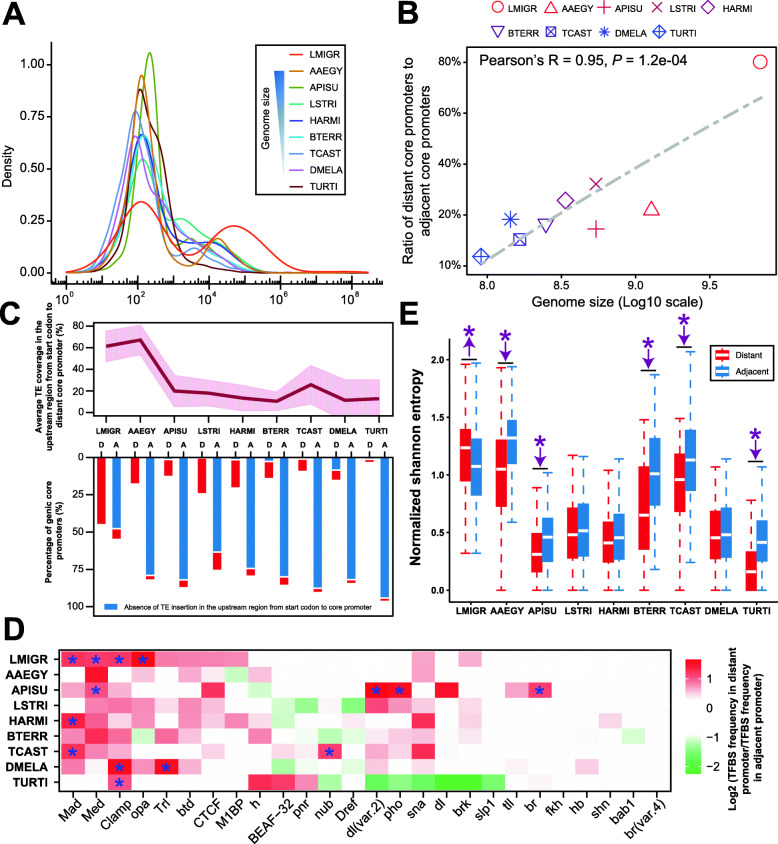
Distant core promoter emergence in the context of genome size evolution in insects. **a** Density distribution of distances from the annotated start codon of protein-coding genes to its farthest core promoters upstream. **b** Correlation between the genome size and the number ratio of distant core promoters to adjacent core promoters. **c** Insertion of TEs in the upstream region from the start codon to core promoter. Top panel: average TE coverage and its standard deviation in the upstream region from the start codon to distant core promoter. Bottom panel: percentage of core promoters that do not contain TEs in the upstream region from the start codon to the distant core promoter. A, adjacent core promoter; D, distant core promoter. **d** TFBS abundance between distant and adjacent core promoters using the number of TFBSs per core promoter per TF. The heatmap was constructed using the log2 transformed ratios of the TFBS abundances between distant and adjacent core promoters. The TFBSs showing significant changes (chi-squared tests with Yates’ correction) in at least one comparison were included in this analysis. Statistical significances were adjusted by Benjamini**–**Hochberg FDR multiple-testing correction. The asterisk indicates a significant difference in the TFBS abundance between adjacent and distant core promoters at a threshold of FDR < 0.01. **e** Estimation of TFBS divergence between distant and adjacent core promoters using normalized SE. The asterisk (*P* < 0.05) indicates a significant difference in the SE value between adjacent and distant core promoters according to the Wilcoxon rank-sum test. *Aedes aegypti*, AAEGY; *Acyrthosiphon pisum*, APISU; *Bombus terrestris*, BTERR; *Helicoverpa armigera*, HARMI; *Laodelphax striatellus*, LSTRI; *Tribolium castaneum*, TCAST; *Tetranychus urticae*, TURTI

To examine the presence of distant core promoters in the context of genome size, we further generated the oligo-capping data from seven arthropod species (six insect species and one chelicerate species), the genome sizes of which are much smaller than the locust genome (Additional file 1: Table S9). The involved insect species, whose genomes represent a wide range of sizes, included *Tribolium castaneum* (red flour beetle, Coleoptera, ~ 0.17 Gb), *Bombus terrestris* (buff-tailed bumblebee, Hymenoptera, ~ 0.25 Gb), *Helicoverpa armigera* (cotton bollworm, Lepidoptera, ~ 0.34 Gb), *Laodelphax striatellus* (small brown planthopper, Hemiptera, ~ 0.54 Gb), *Acyrthosiphon pisum* (pea aphid, Hemiptera, ~ 0.54 Gb), and *Aedes aegypti* (yellow fever mosquito, Diptera, ~ 1.28 Gb). We also included a chelicerate species, *Tetranychus urticae* (two-spotted spider mite, Trombidiformes, ~ 0.09 Gb), as it has the smallest genome size among those of the sequenced arthropod species. The same TSC identification and false TSC removal approaches that were used in the locust data were applied in these arthropod data (Additional file 1: Table S10). The presence of the PyPu dinucleotide initiators guarantees the authenticity of the identified TSCs (Additional file 1: Fig. S25). The genic core promoters, which were linked to the annotated protein-coding genes using paired-read-based assignment rule, showed strong enrichment in the 5′ ends of the gene body (Additional file 1: Fig. S26). Except for the yellow fever mosquito, a unimodal log distribution of the distances from the annotated start codons to the farthest/all genic core promoters upstream was observed in all of the arthropod species, reinforcing our results in the comparison of locusts and fruit flies (Fig. 5a and Additional file 1: Fig. S27). Compared with the migratory locust, the yellow fever mosquito (~ 1.28 Gb) also exhibited a bimodal log distribution with a minor peak shifting towards shorter distances, indicating a lower proportion of distant core promoters in this species. Furthermore, the significant positive correlation between the genome size and the number ratio of distant core promoters to adjacent core promoters suggests that the genome size expansion results in the emergence of distant core promoters to initiate distant transcription (Fig. 5b). Because TEs are the dominant contributors to overall genome size variability in metazoan species [39], we next investigated the contribution of TE insertion into the upstream regions from the start codon to its corresponding core promoter. In the dominant portion of adjacent core promoters, the TE sequences were not detected in the upstream regions from the start codon to the adjacent core promoter, suggesting a strong resistance of TE insertion in adjacent transcription initiation (Fig. 5c, bottom panel). However, in the upstream regions from the start codon to the distant core promoter, the TE sequences could be detected in a large portion of distant core promoters. The decrease in genome size was accompanied by decreases in the average TE coverage in the upstream regions from the start codon to the distant core promoter (Fig. 5c, top panel).

The genomic organization of sequence-specific TFBSs represents a profound recognition source for transcriptional initiation. We evaluated the extent of TF-mediated regulatory elements by analyzing the TFBS occurrence and used the dominant TSSs as the reference point to determine the spatial distribution of TFBSs. TFBSs are enriched from − 125 to 0 bp upstream of the dominant TSS, indicating a positioning bias of TFBSs relative to the TSS (Additional file 1: Fig. S28). To explore the different preferences of sequence context in which the TFBS motif occurs, we compared genome-wide TFBS abundances between adjacent and distant core promoters via the number of TFBSs per core promoter per TF (Fig. 5d). A greater number of TFs showing significantly higher TFBS abundance is observed in the distant core promoters. However, only a few TFs showing significantly higher TFBS abundance are observed in the adjacent core promoters. This suggests that the overall architecture of TF-mediated regulation in distant transcriptional initiation is more variable than that in adjacent transcriptional initiation. To estimate the extent of sequence divergence of TFBSs between the adjacent and distant core promoters, we used a normalized Shannon entropy (SE) as a measure of the degree of conservation of TFBSs for each TF. The higher the SE value is, the higher the sequence divergence of TFBSs. In the migratory locust, the distant core promoters show significantly higher values (*P* < 0.05, Wilcoxon rank-sum test) of SE than adjacent core promoters, suggesting a high variation in TFBSs in distant core promoters (Fig. 5e). However, in the eight other species, the mean values of SE are lower in distant core promoters than in adjacent core promoters. A significantly lower value of SE was observed in five of the eight species involved.

## Discussion

### Exon length constraint of locust genes having distant core promoters

The locust genomic architecture is very different from the architecture of the fly genome. The locust genes are characterized by short exons flanked by long introns. Intron size plays a critical role in determining the splicing efficiency and the recognition mode of the splicing program [40, 41]. The mean intron length in the locust genome is 11.12 kb, which is 12 times longer than the mean intron size (0.88 kb) in the fruit fly genome [20]. Compared with those of locusts, the introns of fruit flies are shorter, with more than half of introns between 40 and 80 bp [42]. Regarding the recognition mode of the splicing program, shorter introns favor intron definition, and longer introns favor exon definition. Therefore, exon definition is the major recognition mode for vertebrate genes, while intron definition is common in lower eukaryotes [43]. The switch from intron definition to exon definition occurs when the intron length is longer than 250 bp [40]. In locust mRNA leaders, only 4.73% of introns have lengths of less than 250 bp, suggesting that the predominant recognition mode in their splicing generally depends on the exon definition model. The intron length distribution in mRNA leaders suggests that the presence of at least one intron in the mRNA leaders results in a transition from adjacent core promoters to distant core promoters in locusts. We found that the exon lengths in the mRNA leaders containing at least one intron in locusts are significantly shorter than those in fruit flies. Notably, the mRNA leaders containing two introns show the least variance in exon length in locusts and that their mean exon length is 118.5 bp. This mean exon length in locusts is consistent with the optimal exon length (~ 150 bp) of the vertebrate exon definition model, which was verified using artificial constructs in previous experimental studies [44]. In contrast, in all of the comparison groups, the mRNA leaders with two introns show the most variance in exon length in fruit flies. This increasing exon length may decrease exon inclusion [45]. These findings suggest that the constraints on the exon length of the mRNA leaders (especially on those derived from distant core promoters) are more important than the constraints on the intron length of the mRNA leaders in the locust genome. In the human genome, short first exons lead to increases in H3K4me3 and H3K9ac at promoters and higher expression levels [46]. This is consistent with the detection of high TSC expression and the strict constraints on exon length in the mRNA leaders with two introns in the distant core promoters of locusts. Taken together, these results suggest that genome size expansion has played a determinant role in the prevalence of splicing recognition modes in locusts and that there is a selection constraint on exon length to preserve optimal splicing effectiveness for distant core promoters in locusts.

### Benefits of distant transcription initiation caused by the presence of large introns

Because the mean intron length in mRNA leaders is 16.11 kb, the presence of at least one intron in the mRNA leaders results in a transition from adjacent core promoters to distant core promoters in locusts. We observed the widespread transcription initiation of distant core promoters, and a considerable proportion of them showed high transcriptional activities, suggesting their functional importance in gene expression. For the protein-coding genes with annotated mRNA leaders, the percentage (32.12%) of the protein-coding genes in which the mRNA leaders contain introns in locusts is greater than that (24.97%) in fruit flies, despite a better annotation of mRNA leaders in fruit flies. This result implies a greater tolerance of introns in mRNA leaders in locusts. Some specific introns in the mRNA leaders have regulatory impact on promoting the transcription and nuclear export of the corresponding host genes [47, 48]. In addition, the enhancer, repressor, and repetitive elements in the introns of mRNA leaders are crucial for transcriptional regulation [49–51]. mRNA leaders containing upstream open reading frames (ORFs) have been identified in approximately one third to half of mRNAs in eukaryotes, and some upstream ORFs have been suggested to play regulatory roles [52]. Furthermore, because splicing order under exon definition does not generally follow the direction of transcription, the distant transcription initiation observed in locusts can overcome the deleterious effects of large intron insertion in mRNA leaders [53]. Considering all of these results together, the regulatory codes of gene expression in locusts may benefit from distant transcription initiation caused by the presence of large introns in mRNA leaders.

### 2 kb limitation rule in promoter function studies of insects

We found that a total of 45.97% of core promoters in locusts are located in the more than 2-kb region upstream of the start codon ATG site. Compared with locusts (~ 6.5 Gb), fruit flies (180 Mb) have a dramatically smaller genome size. Even in the small-sized genome of the fruit fly, a considerable portion (19.41%) of the core promoters are located in the more than 2 kb upstream region of the start codon ATG site, implying a positive correlation between genome size and the distance from the core promoter to the start codon. Because no TSS studies have previously been conducted beyond Diptera, the current catalog of insect core promoters includes only data from this order [8, 10]. The less than 2-kb region upstream of the start codon ATG site has been considered the putative promoter region in a large number of promoter function studies and comparative genomic studies in insects [11–13]. This 2 kb limitation rule has also been used to identify regulatory elements in promoter regions in insects with large genome sizes [14, 54, 55]. However, the alpine grasshopper, *Podisma pedestris*, has the largest genome size identified to date among insects (1C value = 16.93 pg), which is approximately 170-fold larger than the smallest insect genome studied (0.1 pg), indicating that genome sizes vary greatly among insects [56]. These results suggest that researchers should be cautious when the 2 kb limitation rule is applied for promoter function verification, emphasizing the importance of the accurate identification of transcription start sites across diverse insect taxa.

### Imprecise transcription initiation within the core promoter

The increased OTSS diversity within core promoters suggests a less precise transcription initiation of the broad and intermediate core promoters in locusts than in fruit flies. A large majority of the human genome is pervasively transcribed, and protein-coding genes account for only a small proportion of the total transcriptional output [57]. Conversely, little evidence of pervasive transcription has been found in fruit flies [58], implying that genome size differences may contribute to the emergence of pervasive transcription. Compared with fruit flies, both locusts and humans have a genome that is one order of magnitude greater in size. This suggests that the imprecise transcription initiation within core promoters in locusts may be similar in nature to pervasive transcription in humans, in line with the absence of a steep decline in SNP density in the vicinity of locust TSSs. The functionality of the majority of pervasively transcribed transcripts is unknown in humans. Many of these transcripts are thought to be represented as transcriptional noise that is expressed at a low level and results from imprecise transcription initiation due to the promiscuity of RNA polymerase II [59, 60]. However, it has been suggested that cells can benefit from allowing random transcription to occur rather than suppressing nonspecific transcription [61]. Increases in TSC expression are generally achieved by the increasing OTSS diversity within individual core promoters in locusts. These results suggest that imprecise transcription initiation within core promoters does significantly contribute to the activation of protein-coding gene expression in locusts, inconsistent with a role in generating transcriptional noise. However, it is unknown whether OTSS selection to increase OTSS diversity occurs in a random manner or is driven by a specific regulatory manner in locusts.

### Genome size and distant transcription initiation

In the comparison of core promoters across multiple species, the significant positive correlation between the genome size and the number ratio of distant core promoters to adjacent core promoters suggests that the genome size expansion results in the emergence of distant core promoters to initiate distant transcription. However, based on the comparison between locusts and fruit flies, the mRNA leader lengths in the large-sized genome were significantly shorter than those in the small-sized genome in genes having at least one intron in their mRNA leaders. These results suggest that in the large-sized genome, the emergence of distant core promoters was accompanied by increases in the intron size located in mRNA leaders. A large portion of the intergenic region, which is accessible to the transcription machinery, can initiate transcriptional noise at inappropriate positions within intergenic regions [59]. Therefore, transcriptional noise generates long noncoding RNAs, which are usually expressed at a low level [62]. However, a substantial portion of the distant core promoters in locusts showed high transcriptional activity. Furthermore, compared with that in fruit flies, the significantly shorter length of intron-containing mRNA leaders in locusts is consistent with the negative correlation between mRNA leader length and gene expression level [63]. In addition, we linked the distant core promoters in locusts to protein-coding genes using the paired-read-based assignment rule. The results of TFBS abundance and TFBS divergence indicate that the overall architecture of TF-mediated regulation in distant transcriptional initiation is more variable than that in adjacent transcriptional initiation. *Cis*-regulatory elements are composed of motifs that bind to TFs that determine regulatory activity. Thus, the gains and sequence divergence of TFBSs have the potential to modify the regulatory activity of *cis*-regulatory elements. These results imply that as the genome size has expanded, more sophisticated regulatory mechanisms have appeared to drive distant transcription initiation. Taken together, all these results suggest that distant transcription initiation in locusts is not the byproduct of transcriptional noise of lowly expressed long noncoding RNAs in intergenic regions.

## Conclusions

In this study, we generated high-resolution transcription initiation datasets to define a comprehensive atlas of TSCs in locusts, contributing to the expansion of non-*Drosophila* taxonomic representation and revealing distinct genomic features to deepen the understanding of transcription initiation in insects. After conservative computational correction steps, we identified a total of 38,136 reliable TSCs, including 18,305 genic TSCs, in the locust genome. The availability of the locust TSC atlas offers an unprecedented opportunity for the comparative analysis of insect core promoters. The comparison of locust and fruit fly data showed a number of distinct features: the nucleotide composition of the PyPu dinucleotide; the strength and motifs of TATA-box signals; the distribution of the CpG o/e content; the effects of promoter shape on TSC expression specificity, transcription initiation accuracy; and SNP density patterns around core-promoter regions. Furthermore, we revealed a distinctive bimodal log distribution of the distance from the annotated start codons to the core promoters of locust protein-coding genes and defined the adjacent and distant core promoters using a threshold of 3 kb. We found stricter constraints on the exon length of mRNA leaders and widespread higher TSC expression activities of the distant core promoters in locusts compared with fruit flies, implying an important role of distant transcription initiation in locusts. We further compared core promoters in the seven arthropod species across a broad range of genome sizes to reinforce our results on the emergence of distant core promoters in large-sized genomes, and we also revealed the changes in abundance and divergence of TFBSs of distant transcription initiation in the context of genome size.

## Methods

### Insect rearing

The migratory locusts (*Locusta migratoria*) were reared in large, well-ventilated cages (40 cm × 40 cm × 40 cm) at a density of 500–1000 insects per container. These locust colonies were reared under a 14:10 light/dark photoperiod at 30 °C and were fed fresh wheat seedlings and wheat bran.

### RNA isolation, library preparation and sequencing

The tissues and organs were dissected from the fifth-instar nymph of the locust on the third day after molting. Total RNA was isolated using TRIzol reagent (Invitrogen) according to the manufacturer’s instructions. Genomic DNA was removed using TURBO DNase (Invitrogen), and poly-A RNA was enriched twice using a Dynabeads Oligo (dT) 25 kit (Thermo Fisher Scientific). A total of 500 ng of poly-A RNA was treated with calf intestinal alkaline phosphatase (CIP) at 37 °C for 1 h. The reaction mixture was purified using 2.2X Agincourt RNAClean XP beads (Beckman Coulter). The resulting CIP-treated RNA was treated with Tobacco Acid Pyrophosphatase (TAP) at 37 °C for 1 h and purified with 2.2X Agincourt RNAclean XP beads. A 5′ RNA adapter (5′-AGGCACGGGCUAUGAG-3′) was ligated to the CIP/TAP-treated RNA using T4 RNA ligase at 25 °C for 4 h. First-strand cDNA synthesis was carried out using SuperScript IV Reverse Transcriptase (Invitrogen) with 3′ specific random hexamer primers (5′-GATGGAGCGTGTTAGCGNNNNNN-3′). The cDNA was purified with double size selection using 0.6× followed by 0.9× of AMPure XP beads (Beckman Coulter) and was amplified by PCR via Phusion High-Fidelity DNA Polymerase (Thermo Fisher Scientific) in conjunction with 5A (5′-AGGCACGGGCTATGAG-3′) and 3A (5′-GATGGAGCGTGTTAGCG-3′) primers. Finally, sequencing libraries were constructed using the NEBNext Ultra II DNA Library Prep Kit for Illumina (NEB) following the manufacturer’s instructions. A total of 14 oligo-capping libraries were generated and sequenced using the Illumina NovaSeq 6000 System. To generate the oligo-capping data for the seven arthropod species, total RNA was extracted from whole bodies and used for library construction using the protocols as described above.

### Identification of transcription start sites and transcription start site clusters

The raw sequencing reads were preprocessed to remove Illumina adapter sequences and low-quality reads using the Trimmomatic program version 0.36 [64]. The detection of the 5′ oligo-capping and 3′ oligo-capping adapters was achieved using the Cutadapt program version 2.5 [65]. Only the read pairs that contained both the 5′ oligo-capping and 3′ oligo-capping adapters were kept for further analysis. The resulting reads were aligned to the locust genome using the HISAT2 program version 2.1.0 [20, 66]. The soft clipping alignments were not allowed to avoid false TSSs. According to a previous study [23], the PCR duplicates, which were defined as read pairs that share similar alignment coordinates (5′ start of inserts, 3′ end of inserts and splice sites), were removed. The resulting BAM files were used in the identification of oligo-capping transcription start sites (OTSSs) using the CAGEr version 1.24.0 package in R version 3.5.1 and Bioconductor version 3.8 environments [67]. OTSSs with tags per million (TPM) threshold of 3 were used as raw signals for TSS clustering to identify TSCs. The OTSSs, which are separated by less than 20 bp, were clustered into a TSC. To minimize the false TSCs, the TSCs with sequencing reads fewer than 3 TPM were not used in further analysis. The TSC boundaries were calculated based on a cumulative distribution of the sequencing reads to determine the intervals of the 10th and 90th percentiles. TSCs were identified from each library separately. The initial set of TSCs was obtained by merging the TSCs identified from all libraries using a distance threshold of 100 bp. The dominant TSS was defined as the TSS with the highest number of sequencing reads in each TSC. The TSCs mapped to rRNA sequences (28S, 18S, 5.8S, and 5S) were removed from the final set of TSCs.

### Characterization of core promoters of protein-coding genes

Although the TSC is not the same as the promoter, these two terms are used interchangeably in TSS studies because of the strong association between TSCs and promoters [3, 35]. Thus, in this study, the identified TSCs were considered putative core promoters for brevity. The TSCs in the final set were linked to annotated protein-coding genes based on gene structure using paired-end information. For each TSC, if an inserted fragment having its 5′ end in the TSC and its 3′ end in an annotated exon of a protein-coding gene, the TSC was functionally linked to that gene. If a TSC could potentially be tied to multiple protein-coding genes, the TSC was functionally linked to the closest gene. The TSCs showing significant enrichment (observed number greater than 20 and a *q*-value of less than 1e−10) of the TGAG motif and their 1-bp-substitution variants were filtered for further analysis. The significant enrichment motifs were identified using the locust data. The false TSCs derived from internal truncated sites were also filtered for further analyses. The proximal and distal core promoters were defined by comparing two core promoters based on the distances to the annotated start codon, respectively. The core promoter located closer to the downstream start codon of the linked protein-coding gene is considered proximal, whereas the other is considered distal. The adjacent (less than 3 kb) and distant (greater than 3 kb) core promoters were defined based on the distances to the annotated start codon using a threshold of 3 kb, respectively. In intron-level comparison, we did not adopt the rebuilt transcript models that were assembled by the oligo-capping paired-end sequencing reads and the official gene set due to the potential assembly errors in isoform reconstruction [68]. For expression quantification of genic TSCs, the total R1 (forward) reads for each TSC were calculated and normalized to the total library size (defined as the total number of R1 reads derived from any genic TSCs). HOMER version 4.9 was used to perform a de novo motif analysis using the findMotifs.pl tool [69]. RepeatModeler version 2.0.1 was used to generate a de novo repeat library, and the resulting consensus sequences were used to identify genome-wide repeat sequences using RepeatMasker version 4.1.0 [70].

### Expression analyses

As a measure of tissue specificity of TSC expression, *τ* (tau, the tissue specificity index), which is the best metric to measure tissue specificity [33], was calculated using log TPM expression data based on the following equation:

$$ \tau =\frac{\sum_{i=1}^n\left(1-\hat{x_i}\right)}{n-1};\hat{x_i}=\frac{x_i}{\begin{array}{c}\max \left({x}_i\right)\\ {}1\le i\le n\end{array}} $$

The values of *τ* vary from 0 to 1, where 0 < *τ* < 0.1 indicates ubiquitous expression and 0.9 < *τ* < 1 indicates restricted expression.

The biological process enrichment was determined using the clusterProfiler and GO.db packages of the R version 3.5.0 program [71]. The results of GO enrichment analysis were visualized as a scatter plot using the REViGO webserver [72].

### Promoter shape and promoter shifting

The promoter shape was determined using the promoter shape score (PSS), which quantifies the promoter shape based on the promoter width and distribution of oligo-capping reads within a promoter [30]. The PSS was defined by the following equation:

$$ \mathrm{PSS}={\mathrm{Log}}_2w\sum \limits_i^L{p}_i{\mathrm{Log}}_2{p}_i $$

where *p*_*i*_ is the probability of observing an OTSS at base position *i* within a promoter. *L* is the set of base positions that have the normalized TSS expression at a threshold of 3 TPM, and *w* is the promoter width, which is defined as the interval distance between the 10th and 90th quantiles. The PSS values are positively correlated to the width of core promoters, and a PSS value of 0 indicates that all transcription of a core promoter is initiated in a narrow genomic region. The core promoters can be classified into three categories: sharp (PSS > − 10) core promoters, intermediate (PSS ≤ − 10 and PSS > − 20) core promoters, and broad (PSS ≤ − 20) core promoters [30].

The promoter shifting was assessed using the degree of shift (*D*_*s*_ value), which calculates changes in the OTSS distribution between core promoters for each multicore-promoter gene. The *D*_*s*_ value was defined by the following equation:

$$ {D}_S={\mathrm{Log}}_2\left(\frac{P_t/{D}_t}{P_c/{D}_c}\right) $$

where *P*_*t*_ and *D*_*t*_ are the expression abundance values (TPM) of the proximal and distal core promoters in the tested tissue and *P*_*c*_ and *D*_*c*_ are the expression abundance values of the proximal and distal core promoters in the control. A *D*_*s*_ value = 0 indicates the absence of promoter shifting. A *D*_*s*_ value > 1 and a *D*_*s*_ value < −1 indicate promoter shifting towards the proximal and distal core promoters, respectively. To account for multiple comparisons, the raw *P* values from the chi-squared tests were adjusted using the Benjamini–Hochberg method to control the false discovery rate (FDR).

### Nucleotide diversity and OTSS diversity of genic TSCs

The resequencing data of locusts and fruit flies were retrieved from the NCBI Sequence Read Archive (SRA) database under BioProject accessions: PRJNA256231 and PRJNA433455 [37, 38]. The sequencing reads were subject to quality-trimming and were aligned to their corresponding genome using BWA version 0.7.17 with a minimum mapping quality of Q30 [73]. Duplicated reads were filtered, and local realignment and base quality recalibration were performed by GATK version 4–4.1.7. SNPs were identified using the GATK HaplotypeCaller, and raw variants were filtered out based on the following parameters: QD < 2.0 || MQ < 40.0 || FS > 60.0 || SOR > 3.0 || MQRankSum < − 12.5 || ReadPosRankSum < − 8.0 || DP < 10 || DP > 800. To exclude single-nucleotide polymorphism (SNP) calling errors, SNPs showing minor allele frequency greater than 0.5% were kept for subsequent analysis. To determine the nucleotide diversity of the genomic regions flanking TSSs, the 1-kb fragments centered at the TSS were extracted to compute the position-specific density of SNPs.

The Shannon index (*H*) of OTSS diversity for a genic TSC was defined by the following equation:

$$ H=-{\sum}_{i=1}^s{p}_i\mathit{\ln}{p}_i $$

where *S* is the number of OTSSs in a genic TSC and *p*_*i*_ is the proportion of the *i*th OTSS relative to the total number of OTSSs. Following a previous TSS diversity study, the genic TSCs with less than 10 sequencing reads were excluded in Shannon index calculations due to the poor estimation of diversity when the sample size is too small [35]. To remove potential bias caused by different portions of TSCs transcribed from the extremely narrow region between locusts and fruit flies, the genic TSCs with an OTSS number less than 2 were excluded. We performed down-sampling analyses to exclude the potential influences of different TSS profiling methods used and unequal TSC sequencing depths in the comparison between the locust and fruit fly data. We randomly sampled 10 OTSS reads per TSC for the TSCs with at least 10 sequencing reads to generate the down-sampled data.

### Analysis of TFBSs

HOMER version 4.9 was used to perform a de novo motif analysis using the findMotifs.pl tool [69]. Position weight matrices (PWMs) for TFs were taken from DMMPMM (Bigfoot), iDMMPMM, and JASPAR non-redundant insect collection [27]. Find Individual Motif Occurrences (FIMO) in the MEME version 5.0.5 suite was used to determine the occurrences of specific motifs using PWMs with a cut-off value of *P* < 1e− 5 [74]. In TFBS prediction, each PWM was scanned across the − 500 to 100 bp region centered on the dominant TSS in core promoters. Because most of the PWMs used are derived from *Drosophila*, we could not exclude the possibility that these TFs have evolved in different species and thus have modified/alternative TFBS sequence constraints. The predicted TFBSs are considered putative TFBSs because no functional analysis of TFBSs is performed in the arthropod species involved. The statistical significance of the TFBS abundance between adjacent and distant core promoters was calculated using the chi2_contingency function of the Python scipy.stats module. Statistical significances were adjusted by the Benjamini–Hochberg FDR multiple-testing correction. For each TFBS, a normalized Shannon entropy (SE) of multiple sequence alignment was defined to evaluate the TFBS divergence by the following equation:

$$ \mathrm{SE}=-\frac{1}{L}{\sum}_{i=1}^L{\sum}_{i=1}^M{P}_i{Log}_2{P}_i $$

where *P*_*i*_ is the fraction of nucleotide bases of nucleotide base type i, *M* is the number of nucleotide base types, and *L* is the length of the identified TFBS.

## Supplementary Information

**Additional file 1: Figure S1.** Nucleotide composition of OTSSs and nucleotide distribution of sequencing reads. **Figure S2.** Distribution of identified OTSSs in different genomic regions. **Figure S3.** Correlation of the number of tissues involved and the identified OTSSs. **Figure S4.** Distribution of the distance between the identified OTSSs and start codon. **Figure S5.** Width distribution of transcription start site clusters (TSCs) in different genomic regions. **Figure S6.** Consensus 25-bp sequences surrounding the dominant TSSs in different genomic regions. **Figure S7.** A significant enrichment of the TGAG motif and its 1-bp-substitution variants in the 1-bp-wide TSCs. **Figure S8.** Mis-hybridization of the 5′ oligo-capping adaptors and internal RNA sites results in overrepresentation of the TGAG motif. **Figure S9.** False TSCs derived from internal signals in the possible truncated mRNAs. **Figure S10.** Density histogram of the 3′ end of insert fragments along the mRNA transcript with lognormal fit. **Figure S11.** Percentage of removed TSCs by the 3′ end distribution of insert fragments. **Figure S12.** Quantification reproducibility for individual TSCs for two biological replicates. **Figure S13.** Number of identified TSCs per annotated protein-coding gene in the migratory locust and fruit fly. **Figure S14.** Summary of *Drosophila* core promoter elements in the core promoters of locusts and fruit flies. **Figure S15.** CpG distribution in the 4-kb flanking region of transcription start sites. **Figure S16.** Normalized CpG contents of locusts and fruit flies. **Figure S17.** Mean AT contents in the 10 to 50 bp regions upstream of dominant OTSSs of core promoters in locusts and fruit flies. **Figure S18.** Distribution of the tissue-specificity index (tau) of genic TSCs in locusts. **Figure S19.** Scatterplot of enriched GO terms of ubiquitously (Tau = 0) and restricted (tau = 1) TSC expression of locust core promoters. **Figure S20.** Correlation between TSC expression and OTSS diversity via binscatter estimation. **Figure S21.** The alternative usage of core promoters (promoter shifting) in the ovary sample when compared to the muscle sample as a control. **Figure S22.** The alternative usage of core promoters (promoter shifting) of protein-coding genes in different tissue or organ samples when compared to the muscle samples as controls in locusts. **Figure S23.** Distant transcription initiation in locusts and fruit flies. **Figure S4.** Mean intron length in mRNA leaders of locusts and fruit flies. **Figure S25.** Consensus sequences of the 25 bps surrounding the dominant TSSs. **Figure S26.** Meta-profile of TSCs over the gene body of protein-coding genes in the official gene sets. **Figure S27.** The density distribution of distances from the annotated start codon of protein-coding genes to the upstream genic core promoters. **Figure S28.** The abundance distribution of the distances from the TFBSs to the dominant transcription starting site (TSS) in protein-coding genes. **Table S1.** Sequencing data generated in this study for locusts. **Table S2.** Overrepresented motifs of TGAG and its variants in the 1-bp-wide TSCs located in the intergenic region. **Table S3.** Over-represented motifs of TGAG and its variants in the non-1-bp-wide TSCs located in the intergenic region. **Table S4.** Overrepresented motifs of TGAG and its variants in the 1-bp-wide TSCs located in the intronic region. **Table S5.** Over-represented motifs of TGAG and its variants in the non-1-bp-wide TSCs located in the intronic region. **Table S6.** Over-represented motifs of TGAG and its variants in the 1-bp-wide TSCs located in the coding sequence (CDS) region. **Table S7.** Overrepresented motifs of TGAG and its variants in the non-1-bp-wide TSCs located in the coding sequence (CDS) region. **Table S8.** Consensus sequences of *Drosophila* core promoter elements. **Table S9.** Sequencing data generated in this study for the arthropod species. **Table S10.** The identified TSCs in the arthropod species.

## Data Availability

The dataset described or used in this study is available in the NCBI Sequence Read Archive under BioProject accession number PRJNA637188.
