# Supplementary material for: Transcription initiation of distant core promoters in a large-sized genome of an insect
Source: BMC Biol. 2021 Mar 30;19:62. doi: 10.1186/s12915-021-01004-5 (PMC8011201; doi:10.1186/s12915-021-01004-5)
Supplement: Supplementary file 1 — Additional file 1: Figure S1. Nucleotide composition of OTSSs and nucleotide distribution of sequencing reads. Figure S2. Distribution of identified OTSSs in different genomic regions. Figure S3. Correlation of the number of tissues involved and the identified OTSSs. Figure S4. Distribution of the distance between the identified OTSSs and start codon. Figure S5. Width distribution of transcription start site clusters (TSCs) in different genomic regions. Figure S6. Consensus 25-bp sequences surrounding the dominant TSSs in different genomic regions. Figure S7. A significant enrichment of the TGAG motif and its 1-bp-substitution variants in the 1-bp-wide TSCs. Figure S8. Mis-hybridization of the 5′ oligo-capping adaptors and internal RNA sites results in overrepresentation of the TGAG motif. Figure S9. False TSCs derived from internal signals in the possible truncated mRNAs. Figure S10. Density histogram of the 3′ end of insert fragments along the mRNA transcript with lognormal fit. Figure S11. Percentage of removed TSCs by the 3′ end distribution of insert fragments. Figure S12. Quantification reproducibility for individual TSCs for two biological replicates. Figure S13. Number of identified TSCs per annotated protein-coding gene in the migratory locust and fruit fly. Figure S14. Summary of Drosophila core promoter elements in the core promoters of locusts and fruit flies. Figure S15. CpG distribution in the 4-kb flanking region of transcription start sites. Figure S16. Normalized CpG contents of locusts and fruit flies. Figure S17. Mean AT contents in the 10 to 50 bp regions upstream of dominant OTSSs of core promoters in locusts and fruit flies. Figure S18. Distribution of the tissue-specificity index (tau) of genic TSCs in locusts. Figure S19. Scatterplot of enriched GO terms of ubiquitously (Tau = 0) and restricted (tau = 1) TSC expression of locust core promoters. Figure S20. Correlation between TSC expression and OTSS diversity via binscatter estimation. Figure [file 12915_2021_1004_MOESM1_ESM.pdf]

Additional file 1

Transcription initiation of distant core promoters in a large-sized genome of an insect

Qing Liu[1, 2, 3, *], Feng Jiang[1, 4, *], Jie Zhang[1], Xiao Li[5], Le Kang[1, 4, 5, #]

[1] Beijing Institutes of Life Science, Chinese Academy of Sciences, Beijing, China

[2] Sino-Danish College, University of Chinese Academy of Sciences, Beijing, China

[3] Department of Biology, University of Copenhagen, Copenhagen, Denmark

[4] CAS Center for Excellence in Biotic Interactions, University of Chinese Academy of Sciences, Beijing, China

[5] State Key Laboratory of Integrated Management of Pest Insects and Rodents, Institute of Zoology, Chinese Academy of Sciences, Beijing, China

*These authors contributed equally to this study.

Corresponding authors:

Le Kang, Ph.D. and Professor

Institute of Zoology, Chinese Academy of Sciences

Beijing 100101, China

Tel: 86-10-6480-7219

Fax: 86-10-6480-7099

E-mail: lkang@ioz.ac.cn

Supplemental Figures

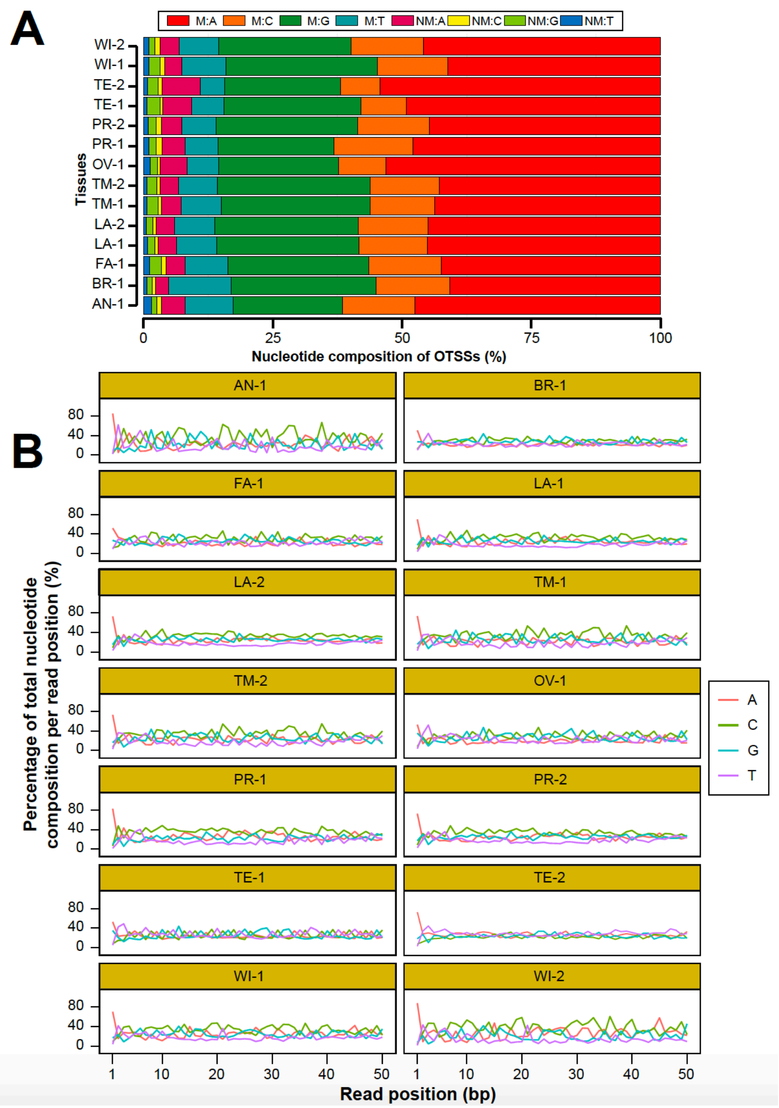

Fig. S1. Nucleotide composition of OTSSs and nucleotide distribution of sequencing reads. (A) The nucleotide composition of OTSSs in the locust genome. M indicates the 5' first-base (of sequencing reads) that is identical to the locust genome in read alignments. NM indicates the 5' first-base (of sequencing reads) that is un-matched to the locust genome in read alignments. (B) The nucleotide distribution of sequencing reads. Ovary, OV; testis, TE; wing, WI; thoracic muscle, TM; pronotum, PR; labipalp, LA; brain, BR; fat body, FA; antenna, AN.

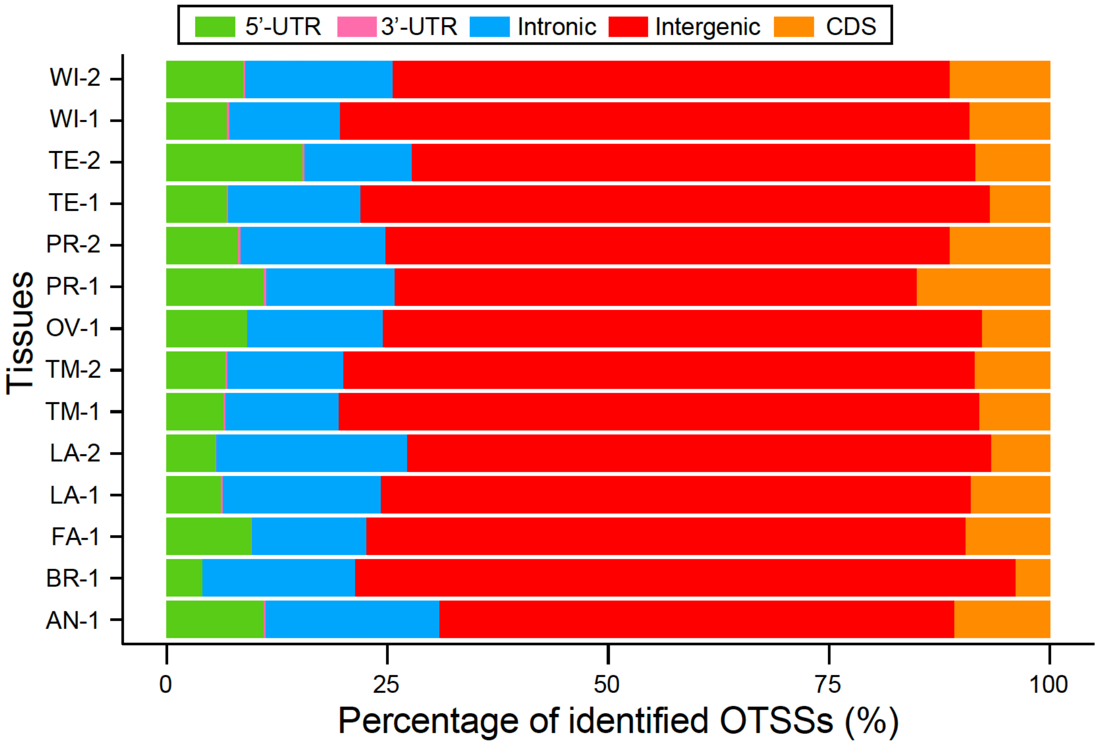

Fig. S2. Distribution of identified OTSSs in different genomic regions. CDS, coding sequences; UTR, untranslated region; ovary, OV; testis, TE; wing, WI; thoracic muscle, TM; pronotum, PR; labipalp, LA; brain, BR; fat body, FA; antenna, AN.

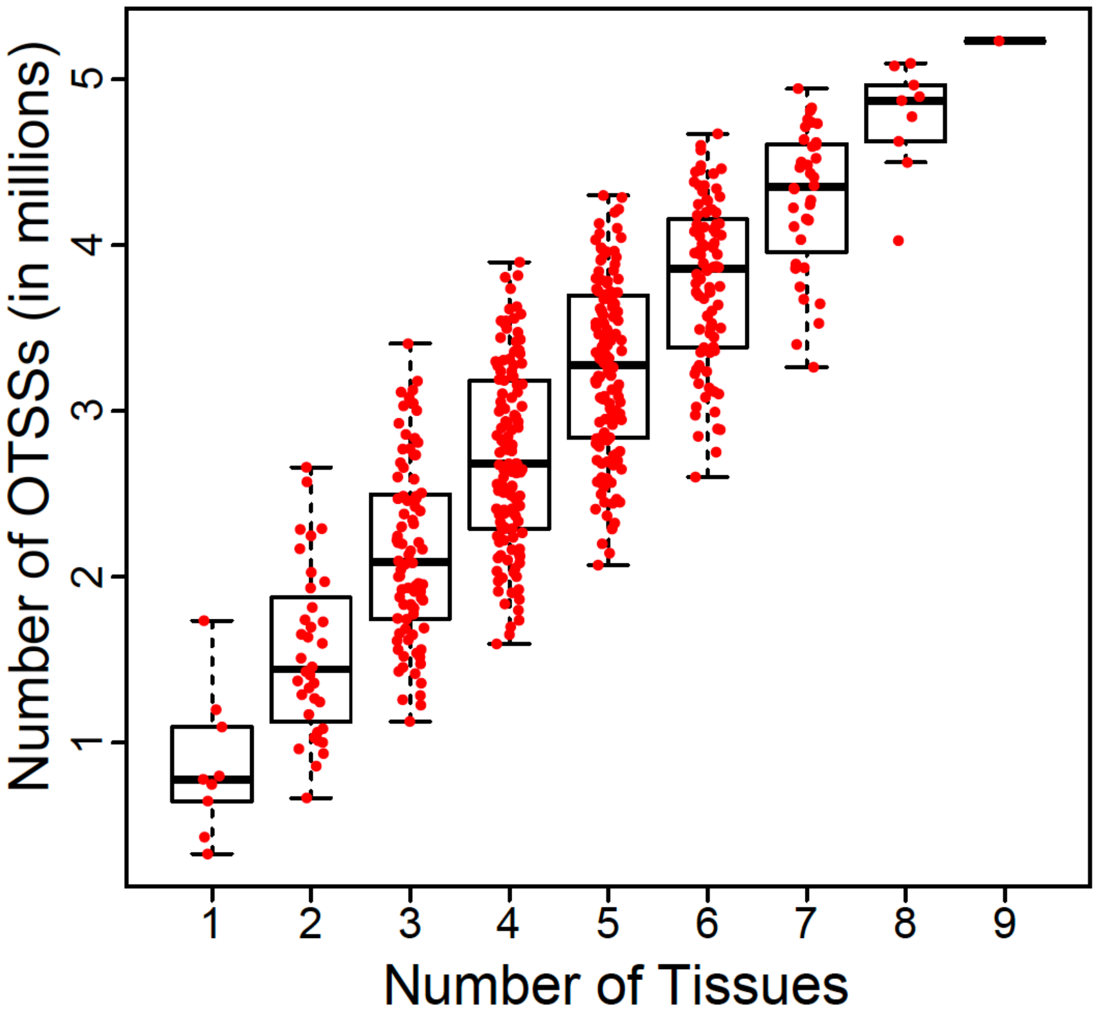

Fig. S3. Correlation of the number of tissues involved and the identified OTSSs. The x-axis shows the number of tissues involved. The red dots in the box plots represents the identified OTSSs based on a combination of OTSS data in the number (ranging from 1 to 9) of the tissues involved.

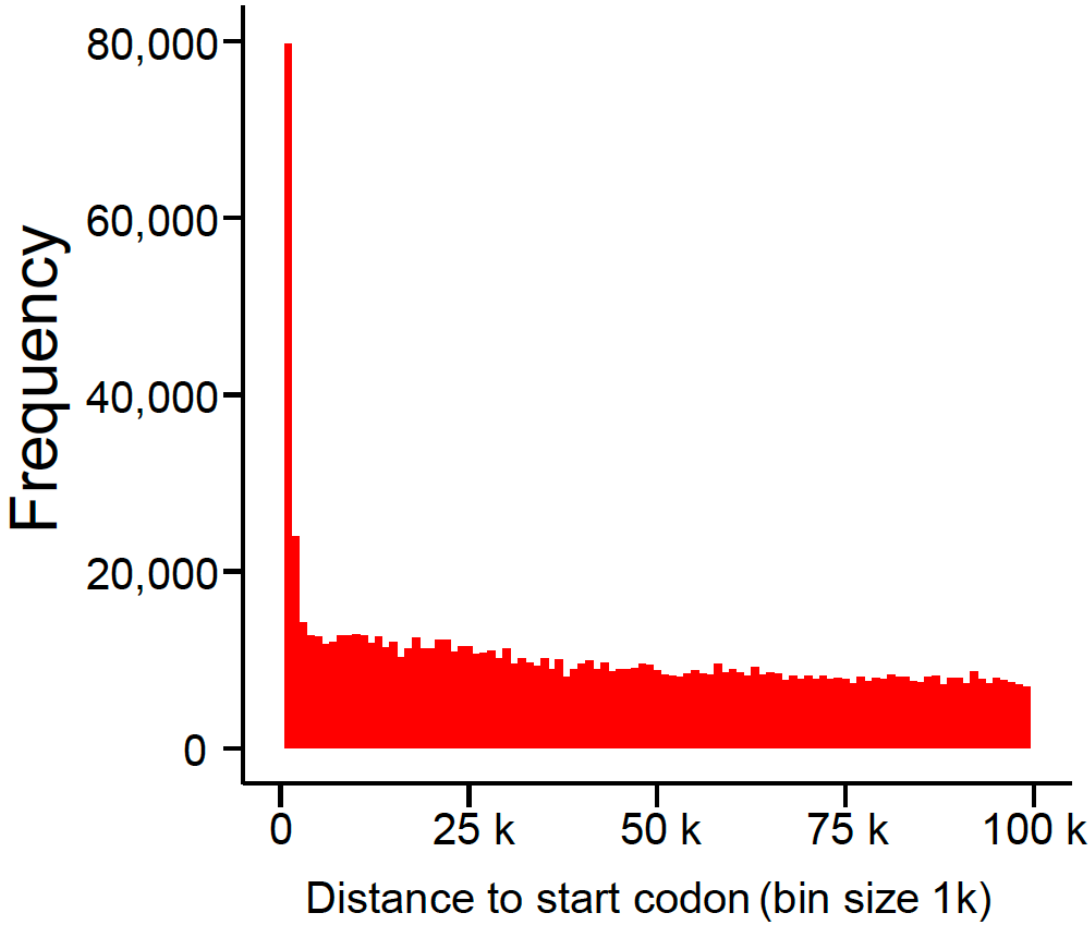

Fig. S4. Distribution of the distance between the identified OTSSs and start codon. The distance was calculated as the distance from the start codons of protein-coding genes to the identified OTSSs upstream along the locust genome.

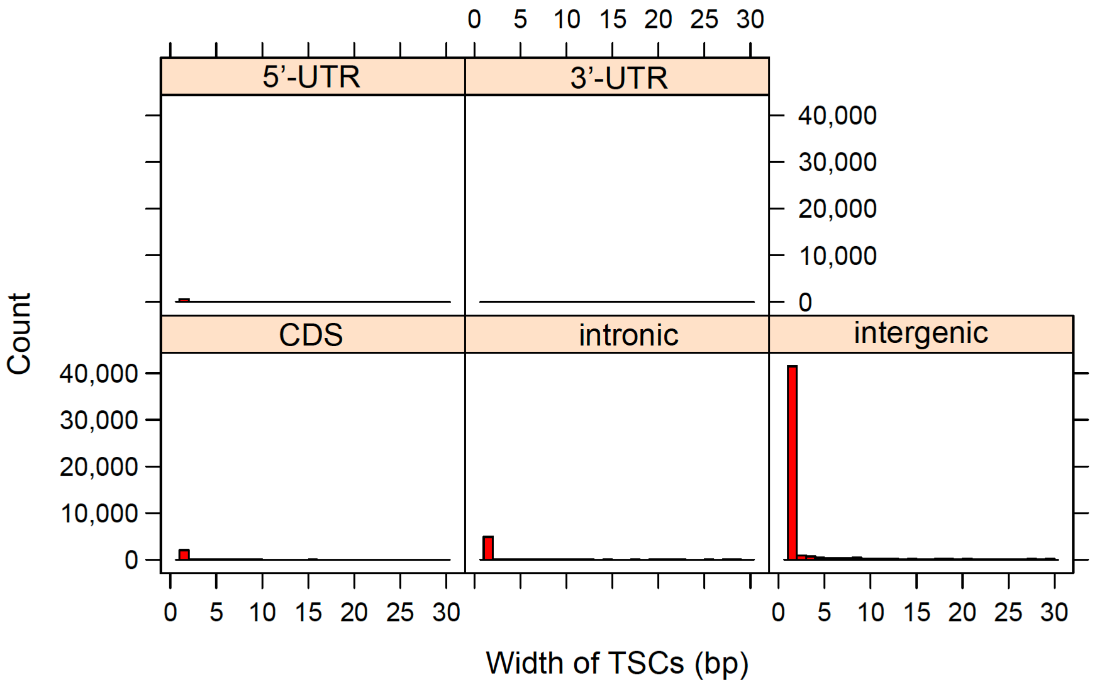

Fig. S5. Width distribution of transcription start site clusters (TSCs) in different genomic regions. Only the TSCs that are less than 30 bp in width are included. CDS, coding DNA sequence; UTR, untranslated region.

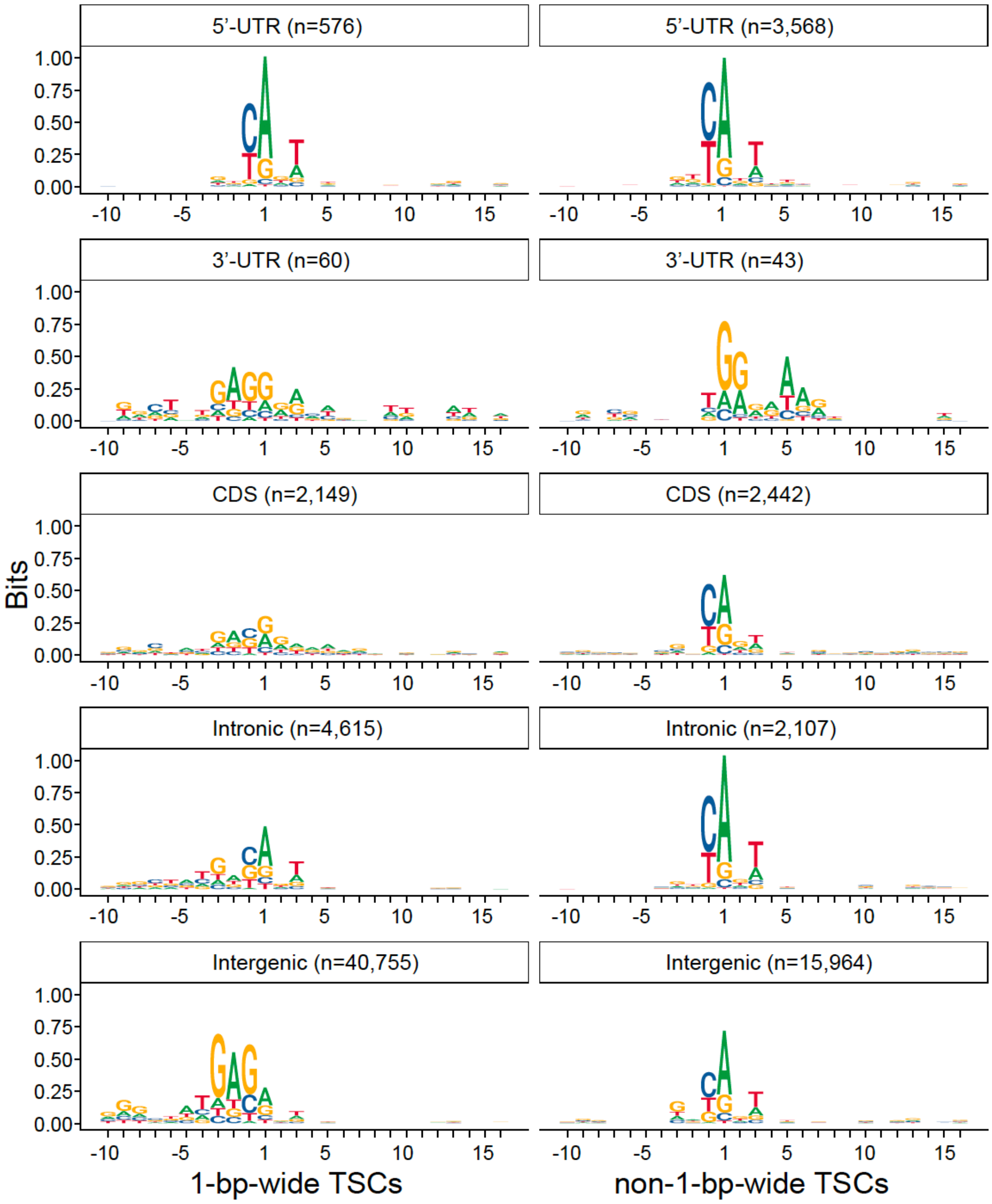

Fig. S6. Consensus 25-bp sequences surrounding the dominant TSSs in different genomic regions. The symbol height within the stack indicates the relative frequency of each nucleic acid at that position. The frequency of each nucleotide for each position was represented using the R package Seqlogo.

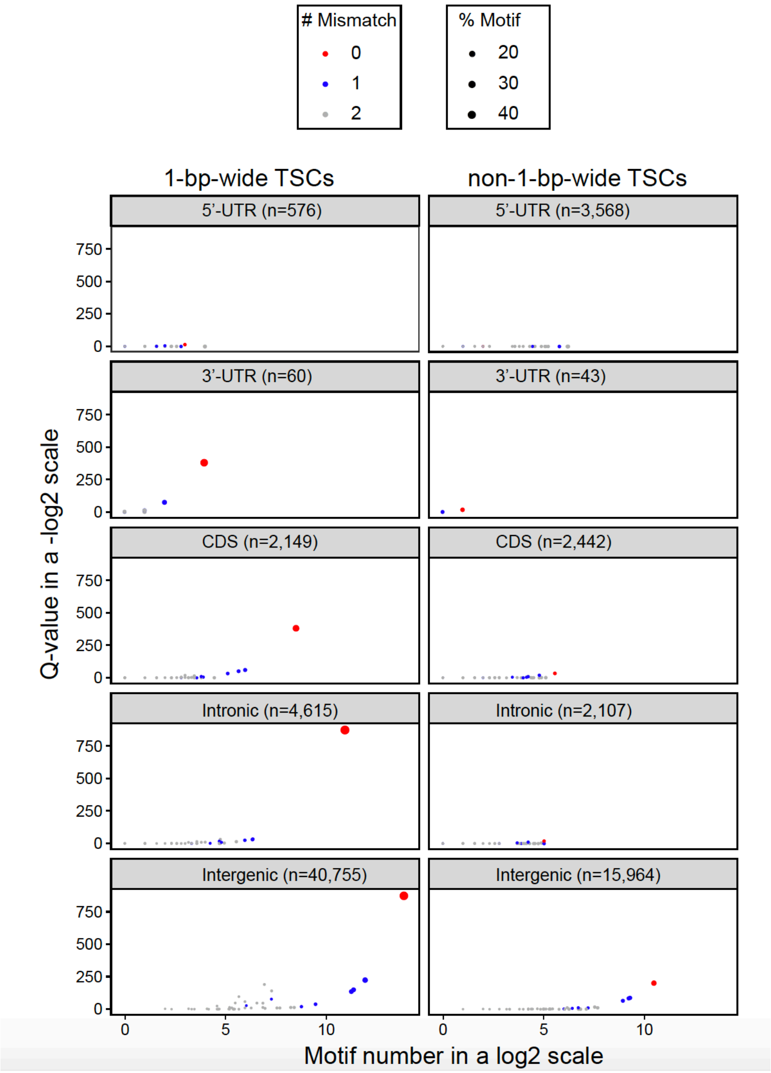

Fig. S7. A significant enrichment of the TGAG motif and its 1-bp-substitution variants in the 1-bp-wide TSCs. The significance of overrepresented motifs in the 1-bp-wide TSCs located in the intergenic region was tested using Fisher's exact test, which was applied by the qvalue package in R. "# Mismatch" represents the substitution number compared to the TGAG motif. "% Motif" represents the percentage relative to the total number of the 1-bp-wide TSCs in each genomic region.

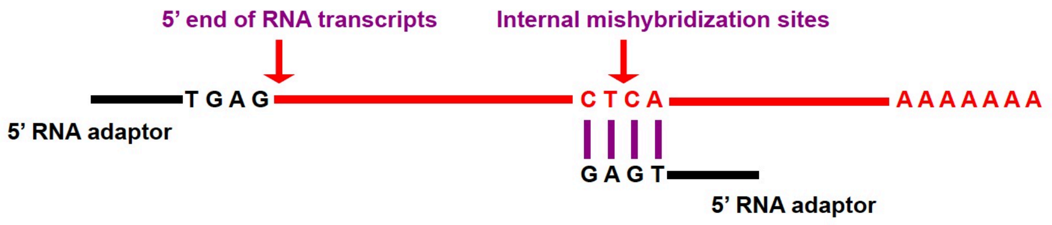

Fig. S8. Mis-hybridization of the 5’ oligo-capping adaptors and internal RNA sites results in overrepresentation of the TGAG motif.

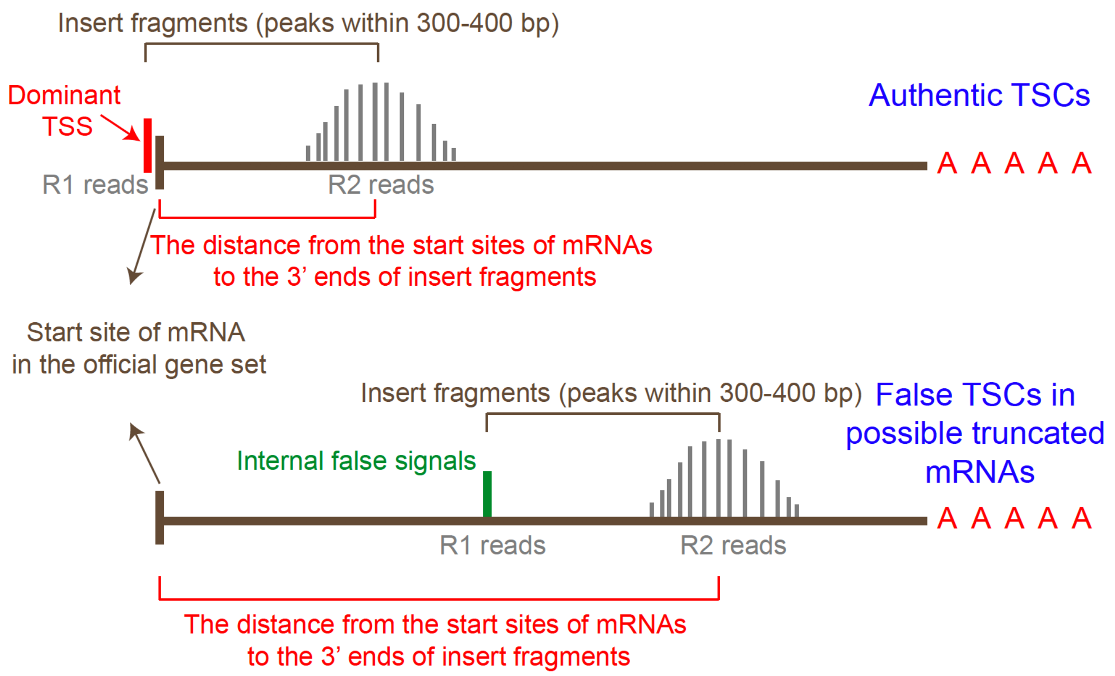

Fig. S9. False TSCs derived from internal signals in the possible truncated mRNAs. The distance from the start sites of mRNAs to the 3’ ends of insert fragments was inferred by determining the start sites of paired R2 (reverse) reads.

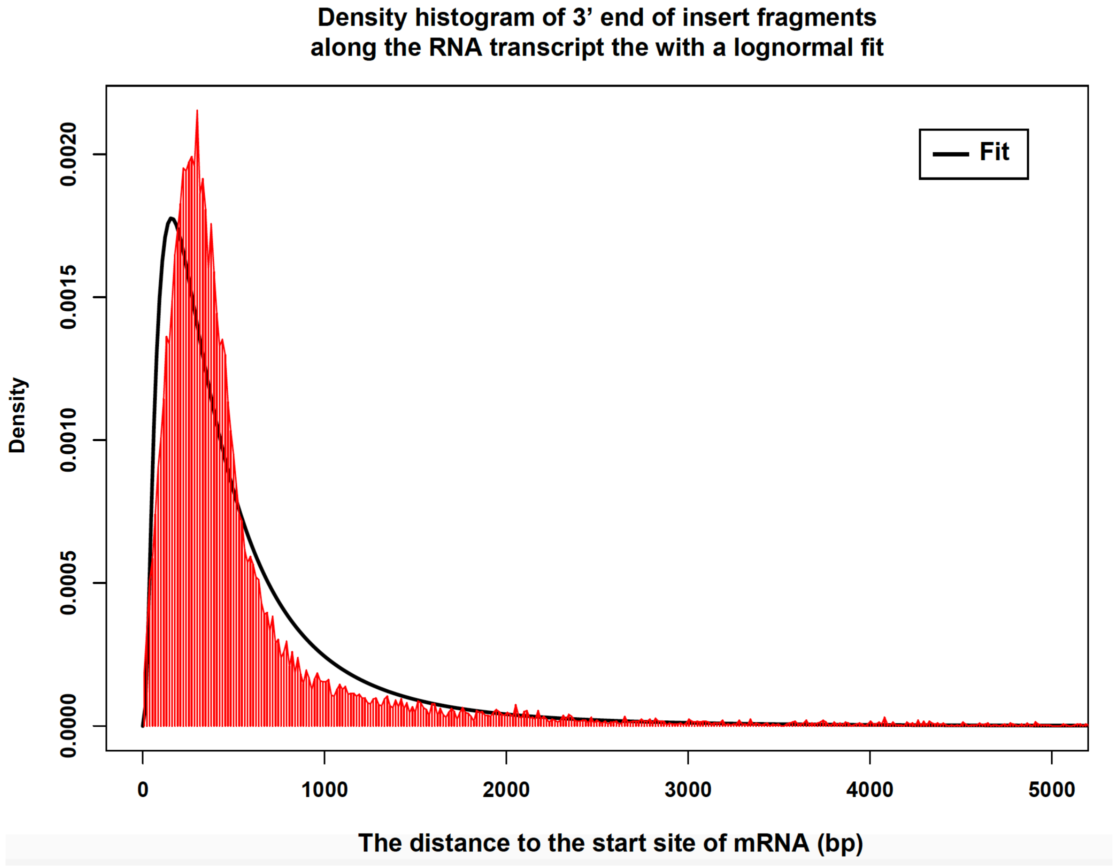

Fig. S10. Density histogram of the 3' end of insert fragments along the mRNA transcript with lognormal fit. The 3' end of the insert fragment along the RNA transcript (in red) was inferred by determining the start sites of R2 (reverse) reads.

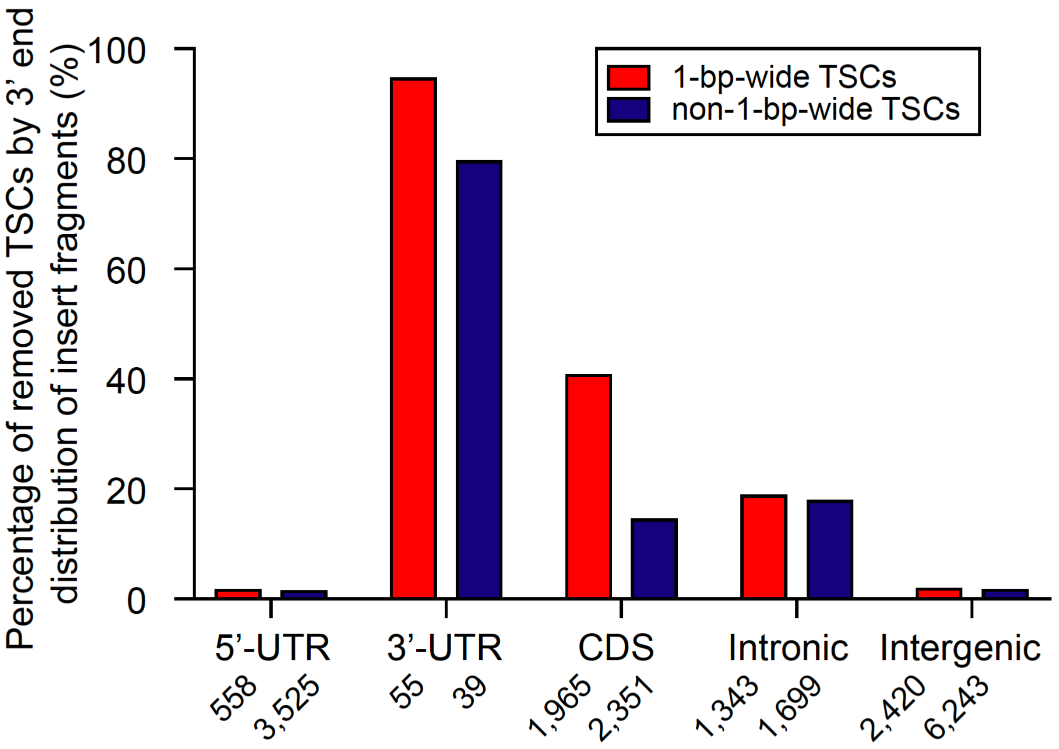

Fig. S11. Percentage of removed TSCs by the 3' end distribution of insert fragments. The numbers on the x-axis represent the total number of TSCs in each genomic region.

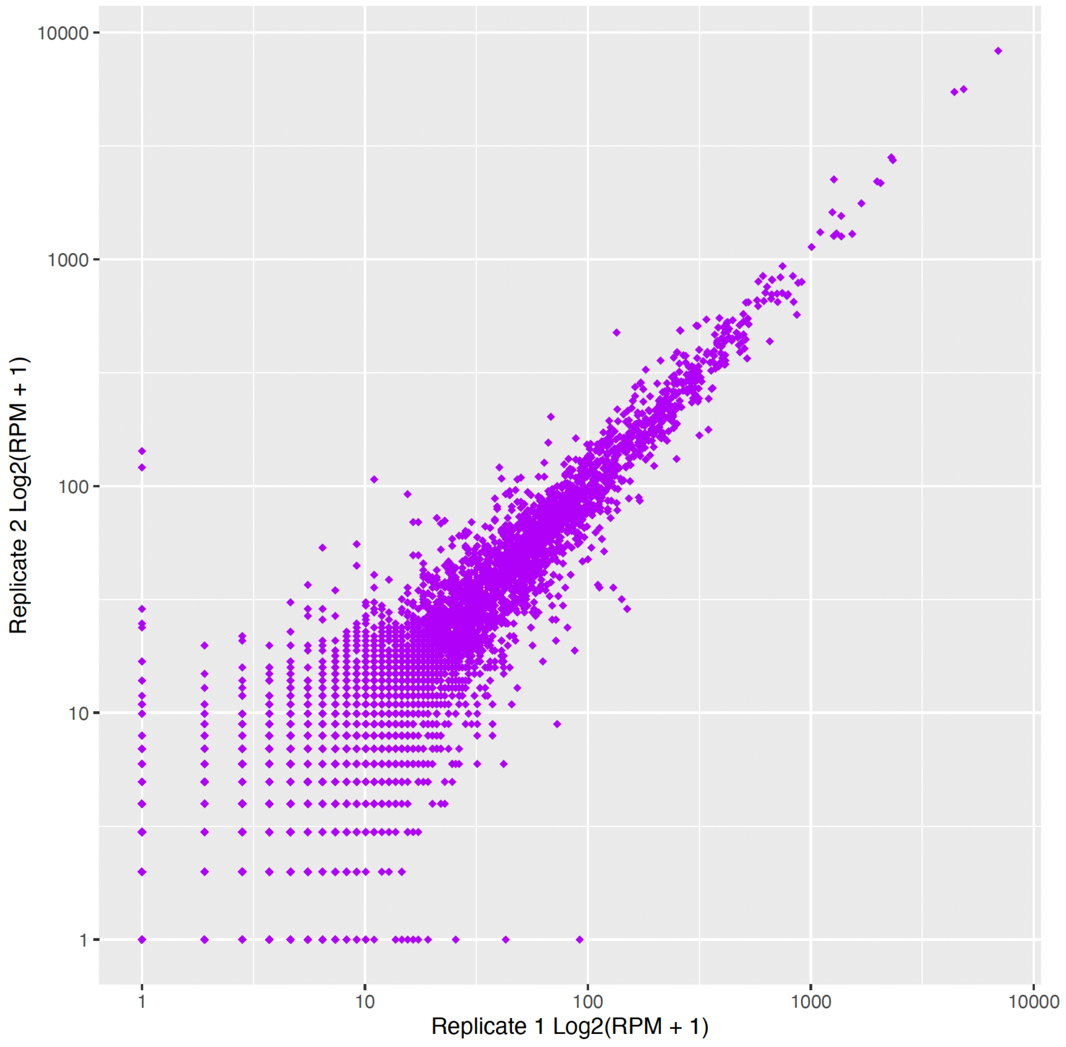

Fig. S12. Quantification reproducibility for individual TSCs for two biological replicates.

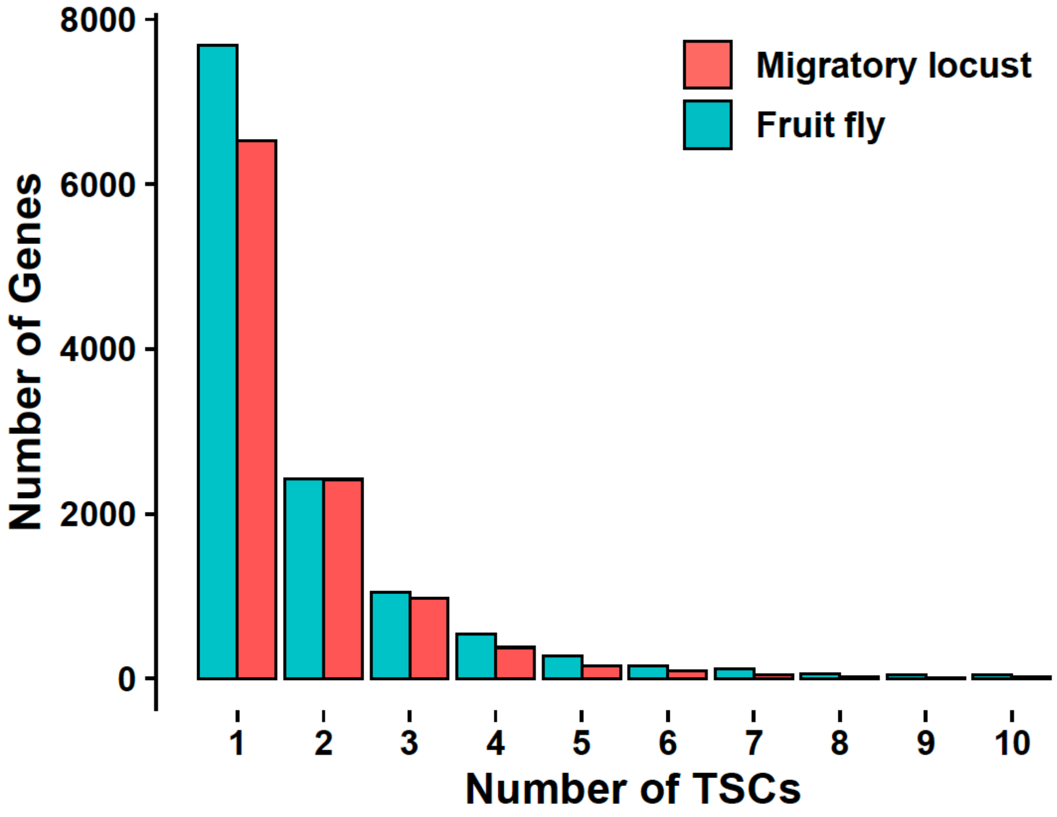

Fig. S13. Number of identified TSCs per annotated protein-coding gene in the migratory locust and fruit fly.

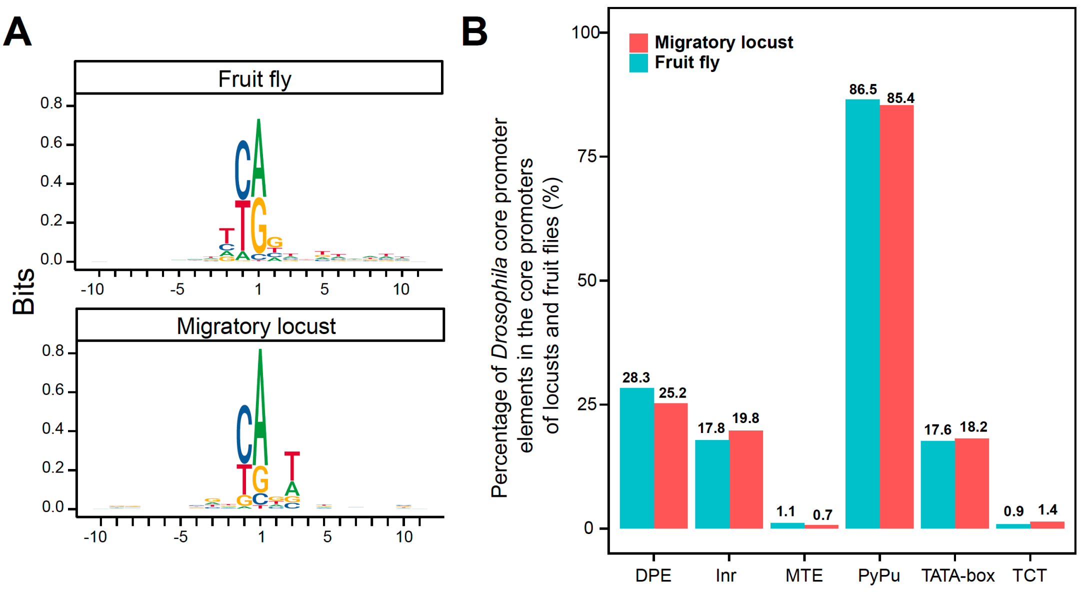

Fig. S14. Summary of *Drosophila* core promoter elements in the core promoters of locusts and fruit flies. (A) The nucleotide composition flanking initiators of genic TSCs in locusts and fruit flies. A preference of T/A usage in the 2 bp downstream of initiators was observed in the genic core promoters of locusts. Although the *Drosophila* Inr is TCA+1GTY in the current consensus, it contributes only a minor portion of the putative Inr motifs in *Drosophila* promoters. The PyPu dinucleotide is probably the most commonly occurring motif of initiator elements throughout eukaryotes from yeasts to bilaterian animals. Therefore, the PyPu dinucleotide is used in the characterization and assessment of genic TSCs in this study. (B) Summary of *Drosophila* core promoter elements in the core promoters of locusts and fruit flies. The consensus sequences were used in pattern matching of the putative *Drosophila* core promoter elements while allowing one mismatch. Inr, initiator; TCT, polypyrimidine initiator; MTE, motif ten element; DPE, downstream core promoter element.

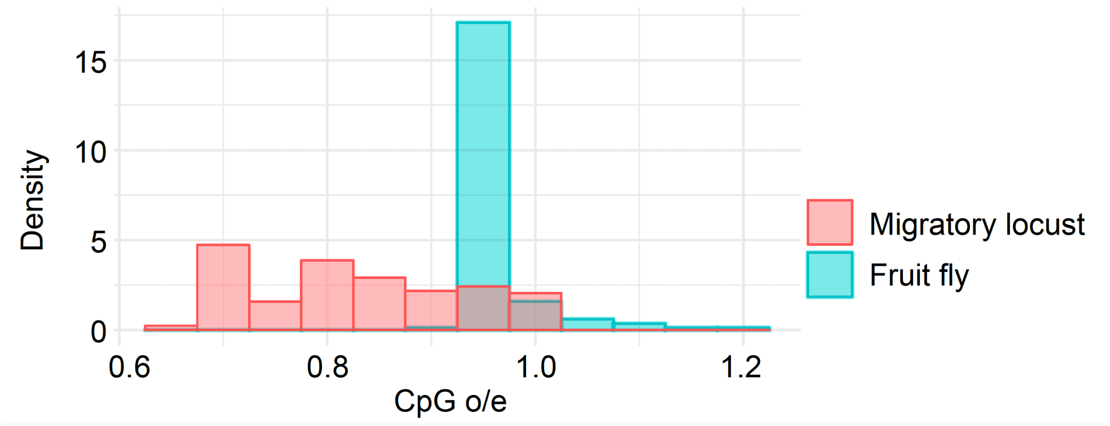

Fig. S15. CpG distribution in the 4-kb flanking region of transcription start sites.

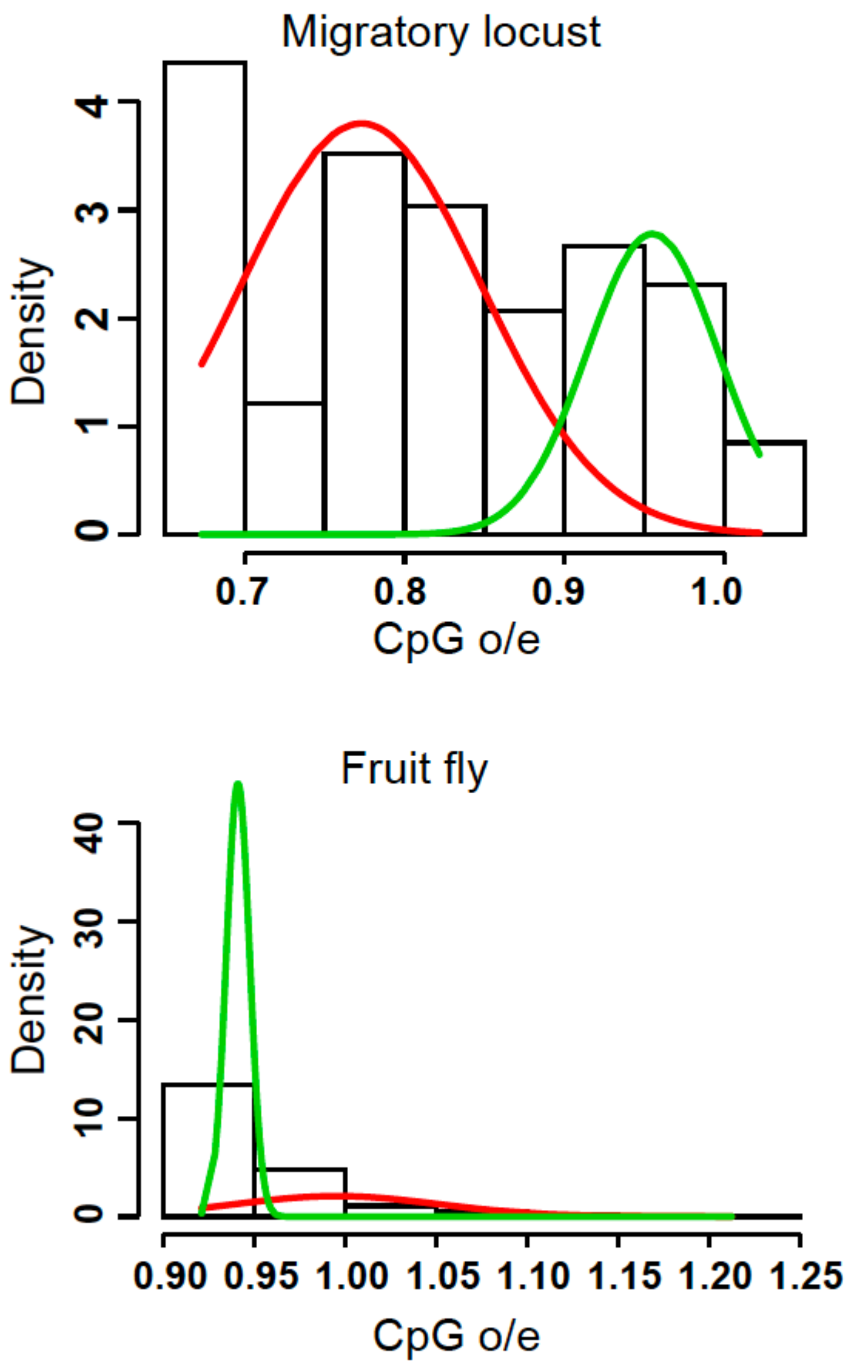

Fig. S16. Normalized CpG contents of locusts and fruit flies. Distributions of normalized CpG contents (CpG observed/expected, CpG oe) with a mixture of two normal distributions fitted to the CpG data using the Mixtools package in R.

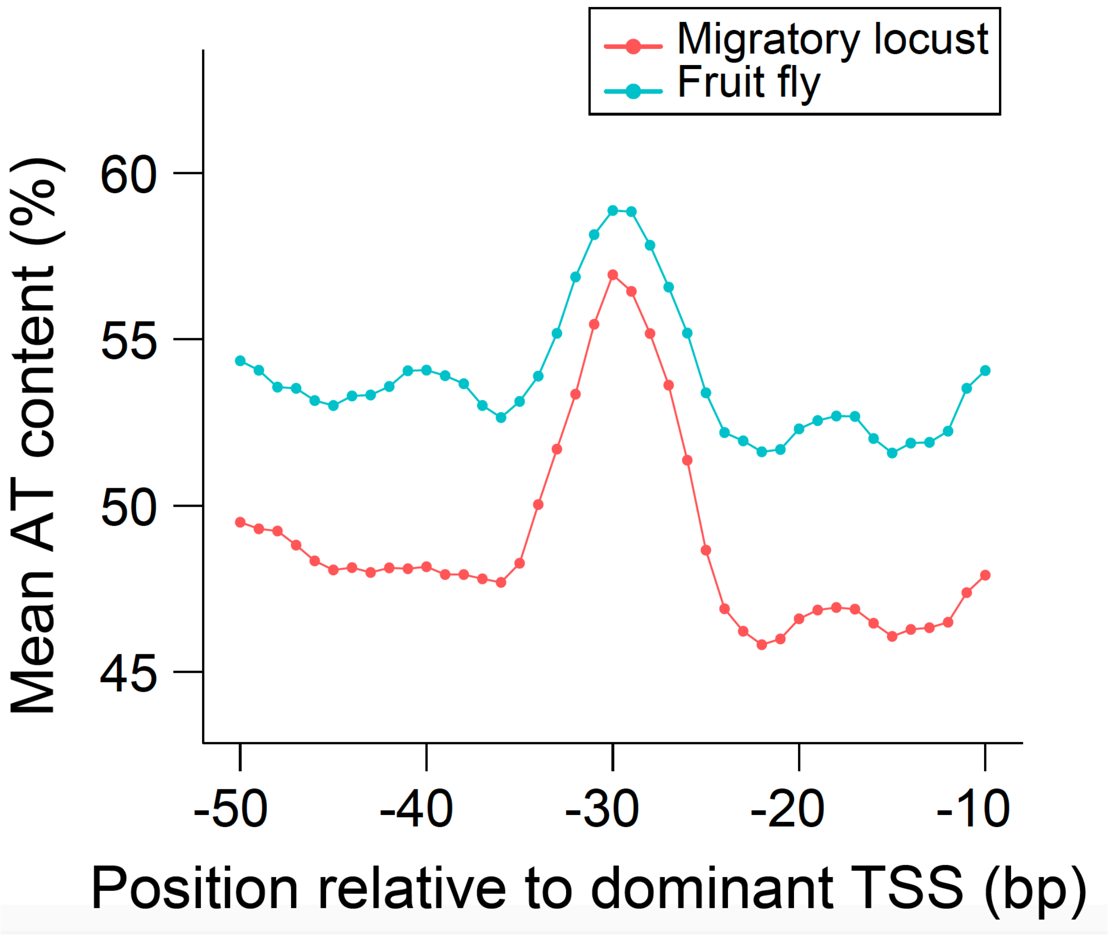

Fig. S17. Mean AT contents in the 10 to 50 bp regions upstream of dominant OTSSs of core promoters in locusts and fruit flies. The mean AT contents were determined using a sliding windows analysis.

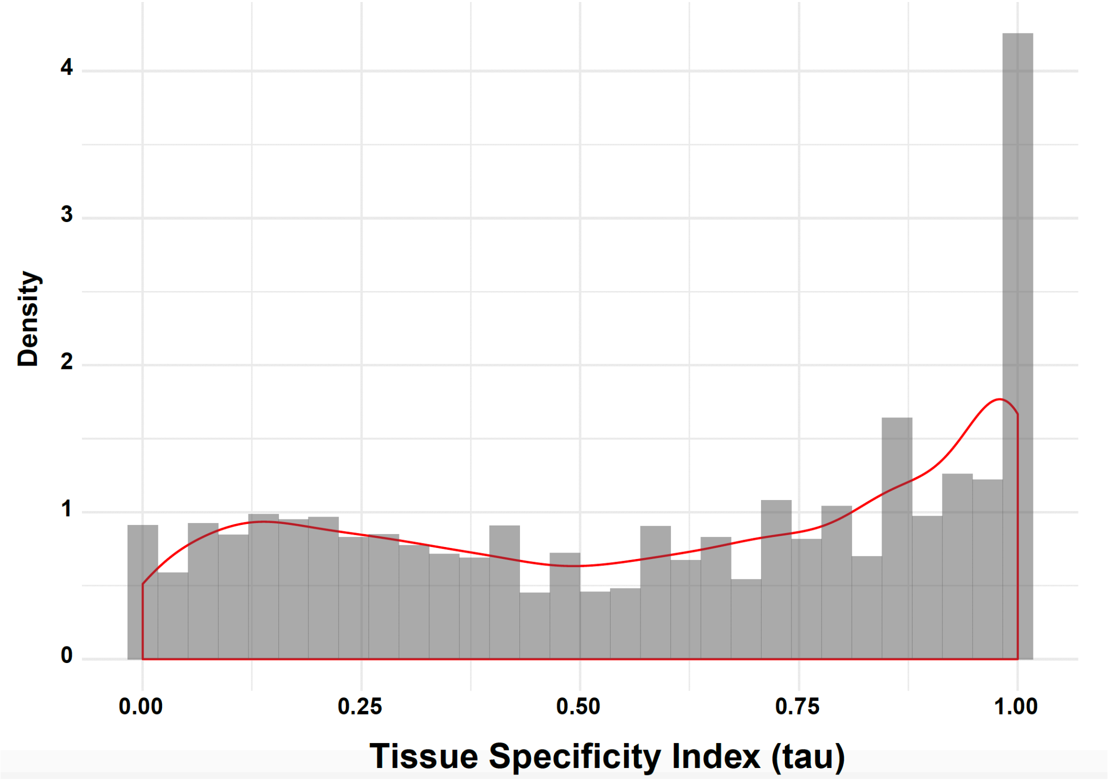

Fig. S18. Distribution of the tissue-specificity index (tau) of genic TSCs in locusts. The testis data was not included in the tissue-specificity analysis due to the testis-specificity of widespread expression.

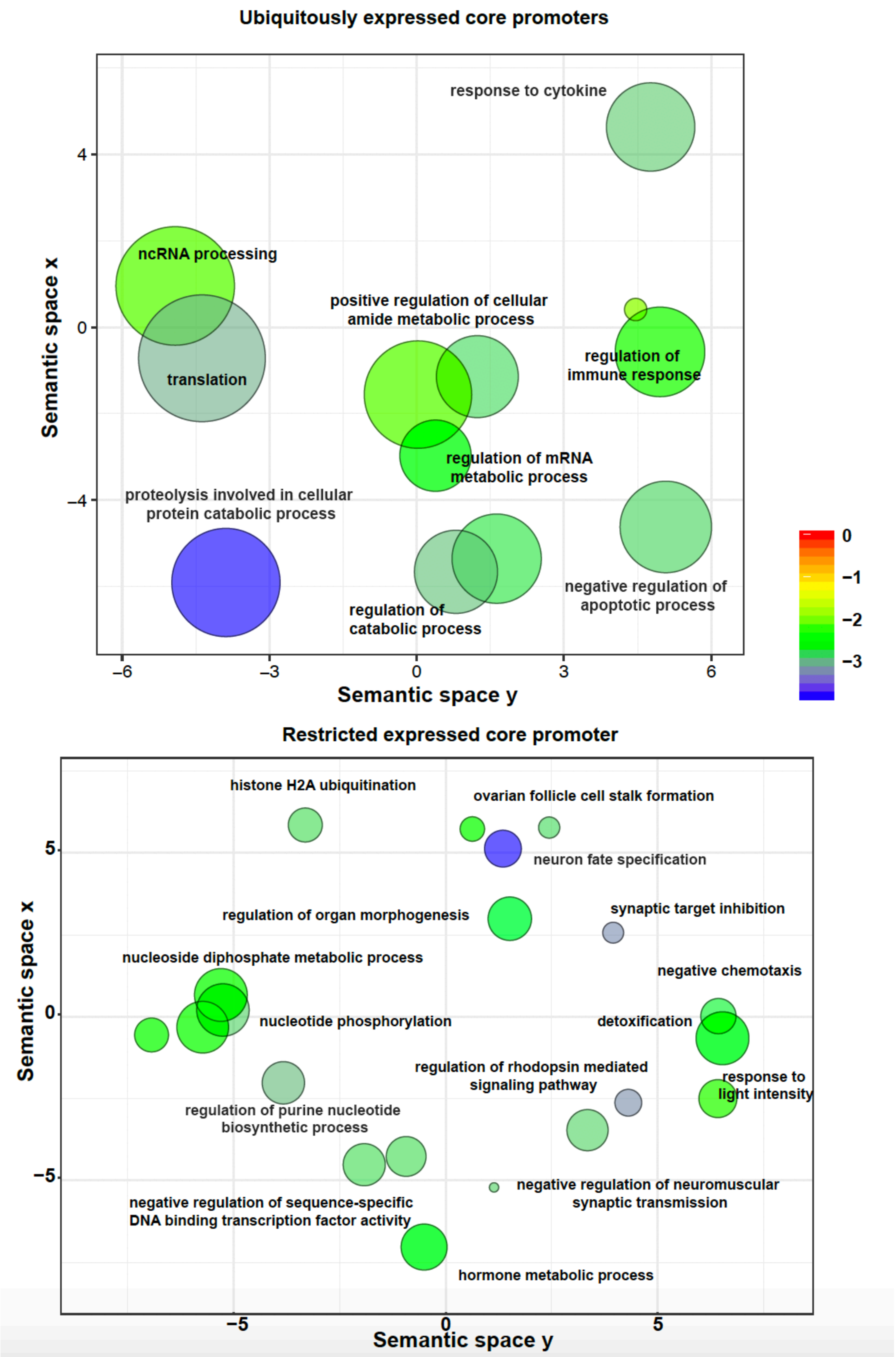

Fig. S19. Scatterplot of enriched GO terms of ubiquitously (Tau = 0) and restricted (tau = 1) TSC expression of locust core promoters.

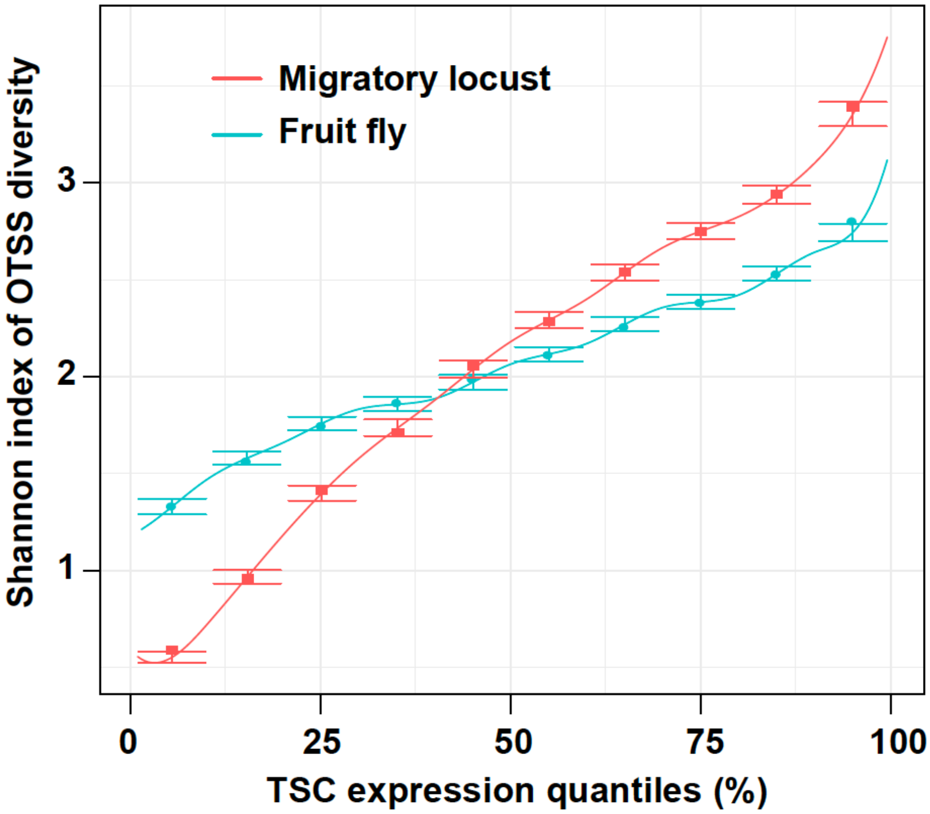

Fig. S20. Correlation between TSC expression and OTSS diversity via binscatter estimation.

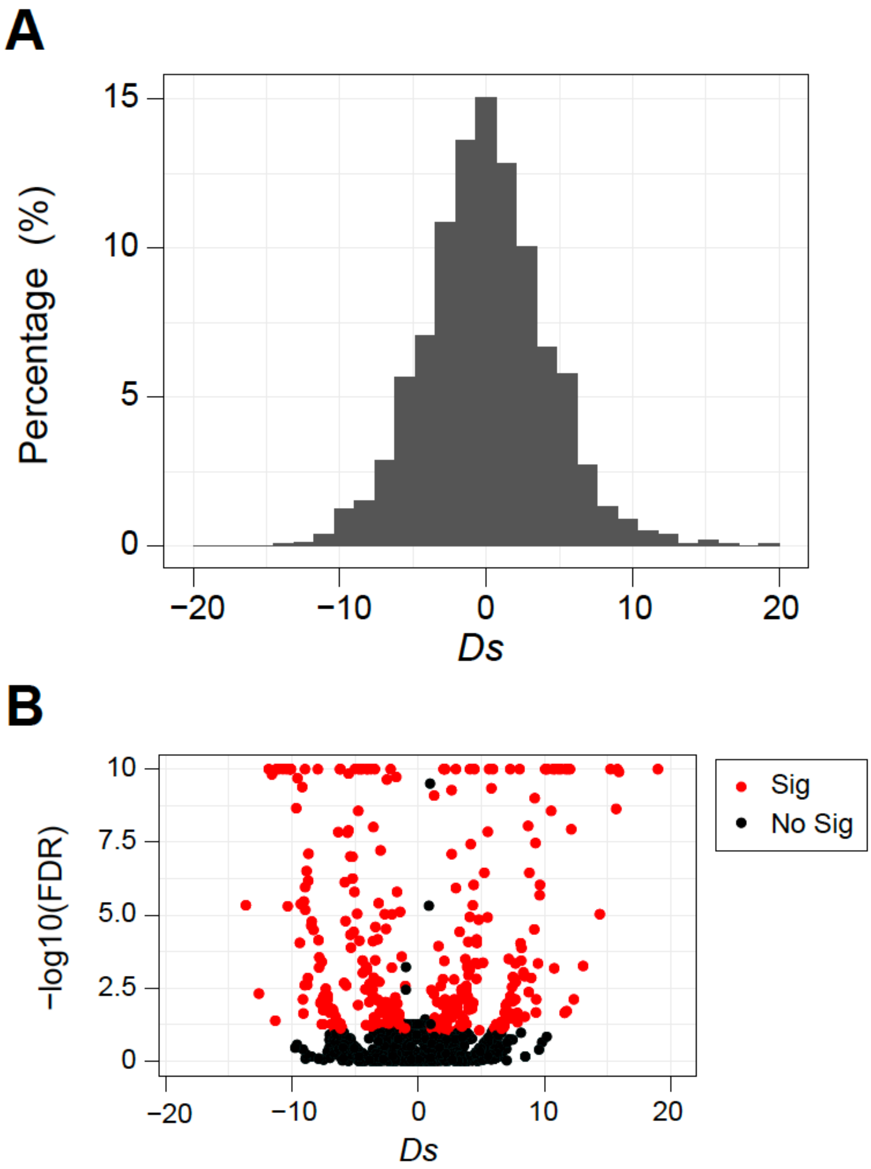

Fig. S21. The alternative usage of core promoters (promoter shifting) in the ovary sample when compared to the muscle sample as a control. (A) Distribution of *Ds* values. (B) Volcano plot showing the *Ds* values and −log10 FDR values. The dots in red represent the protein-coding genes with a significant (Sig) promoter shift ($P < 0.05$, chi-squared tests; FDR < 0.1 and $Ds < -1$ or $Ds > 1$).

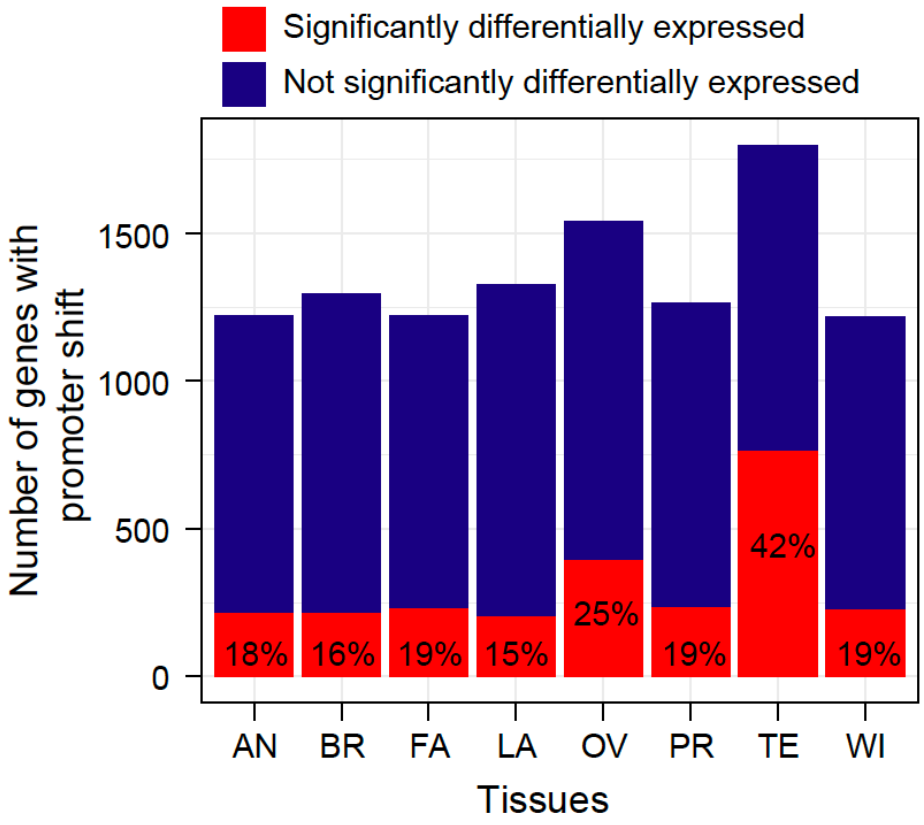

Fig. S22. The alternative usage of core promoters (promoter shifting) of protein-coding genes in different tissue or organ samples when compared to the muscle samples as controls in locusts. Ovary, OV; testis, TE; wing, WI; thoracic muscle, TM; pronotum, PR; labipalp, LA; brain, BR; fat body, FA; antenna, AN.

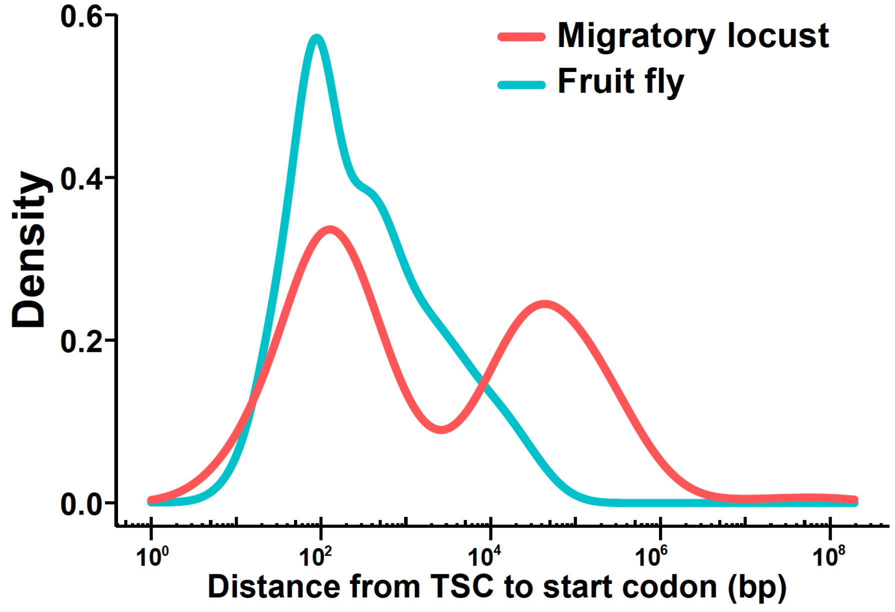

Fig. S23. Distant transcription initiation in locusts and fruit flies. The density distribution of distances from the annotated start codon of protein-coding genes to the upstream genic core promoters.

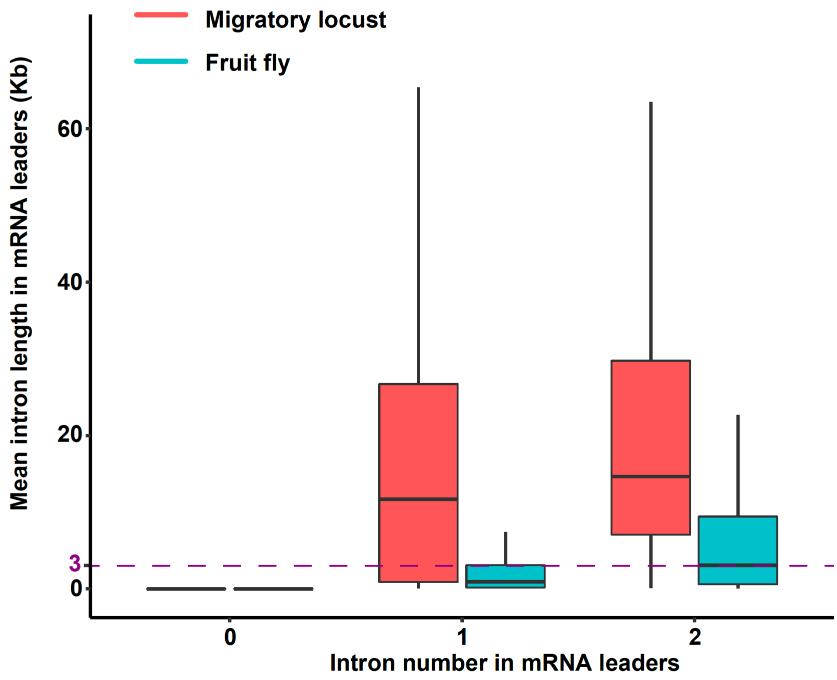

Fig. S24. Mean intron length in mRNA leaders of locusts and fruit flies. Since the length of the first intron is longer than the other ones in complex eukaryotes, the mean intron lengths were used in this comparison.

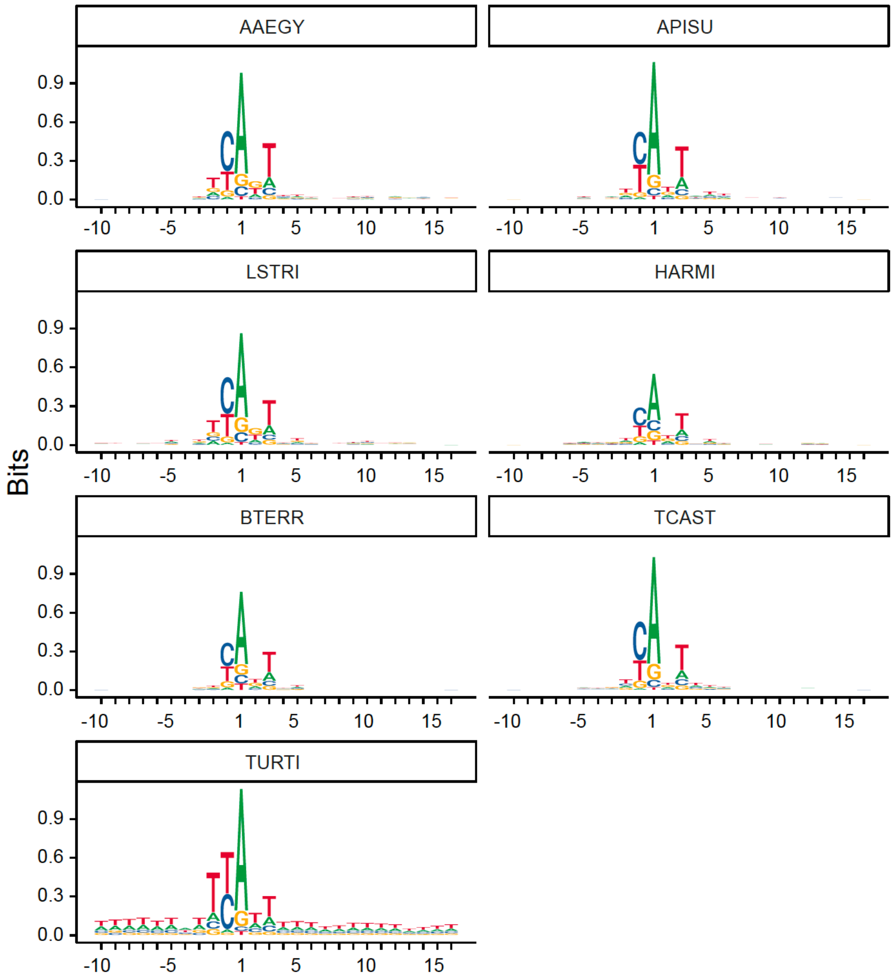

Fig. S25. Consensus sequences of the 25 bps surrounding the dominant TSSs. The symbol height within the stack indicates the relative frequency of each nucleic acid at that position. The frequency of each nucleotide for each position was represented using the R package Seqlogo. *Aedes aegypti*, AAEGY; *Acyrthosiphon pisum*, APISU; *Bombus terrestris*, BTERR; *Helicoverpa armigera*, HARMI; *Laodelphax striatellus*, LSTRI; *Tribolium castaneum*, TCAST; *Tetranychus urticae*, TURTI.

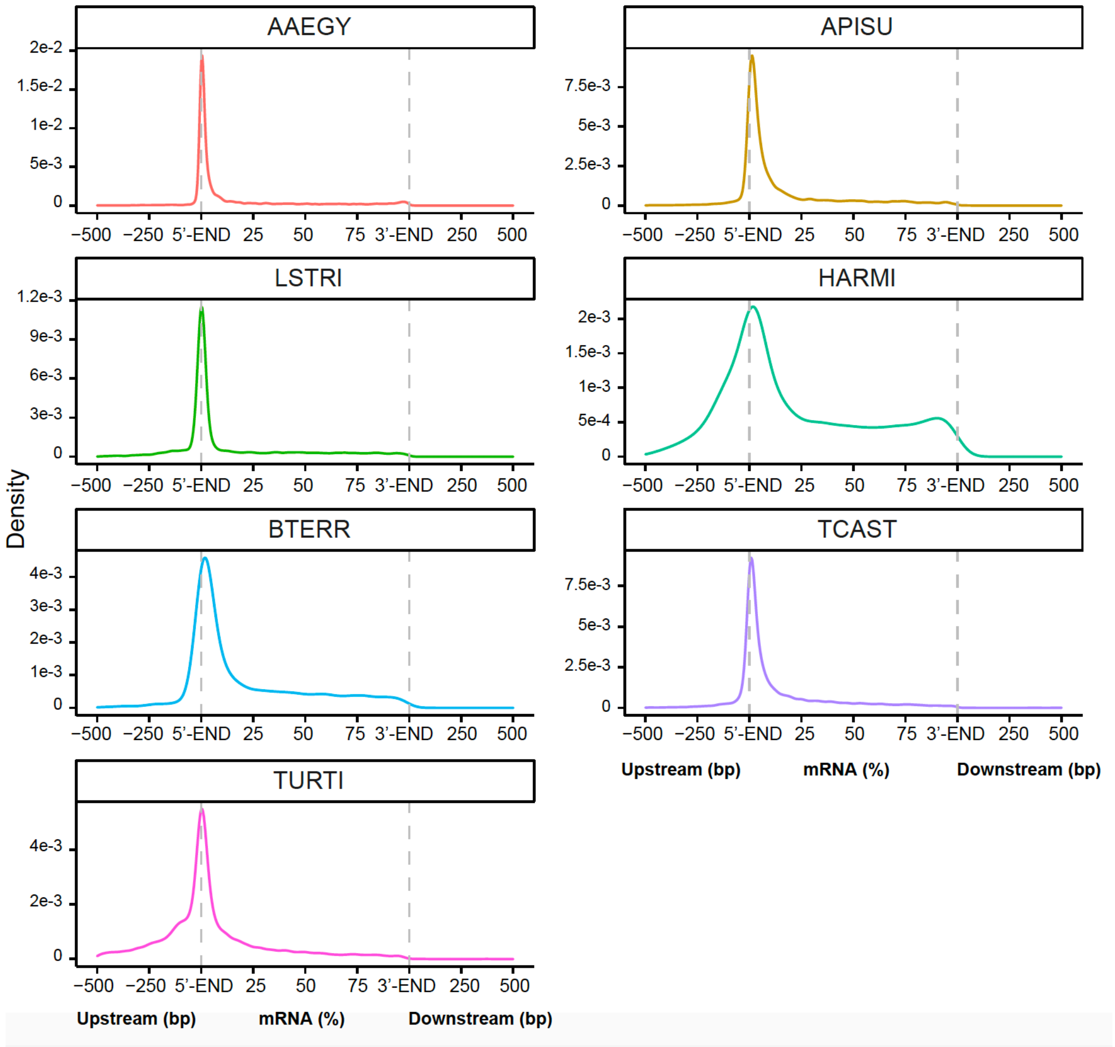

Fig. S26. Meta-profile of TSCs over the gene body of protein-coding genes in the official gene sets. *Aedes aegypti*, AAEGY; *Acyrthosiphon pisum*, APISU; *Bombus terrestris*, BTERR; *Helicoverpa armigera*, HARMI; *Laodelphax striatellus*, LSTRI; *Tribolium castaneum*, TCAST; *Tetranychus urticae*, TURTI.

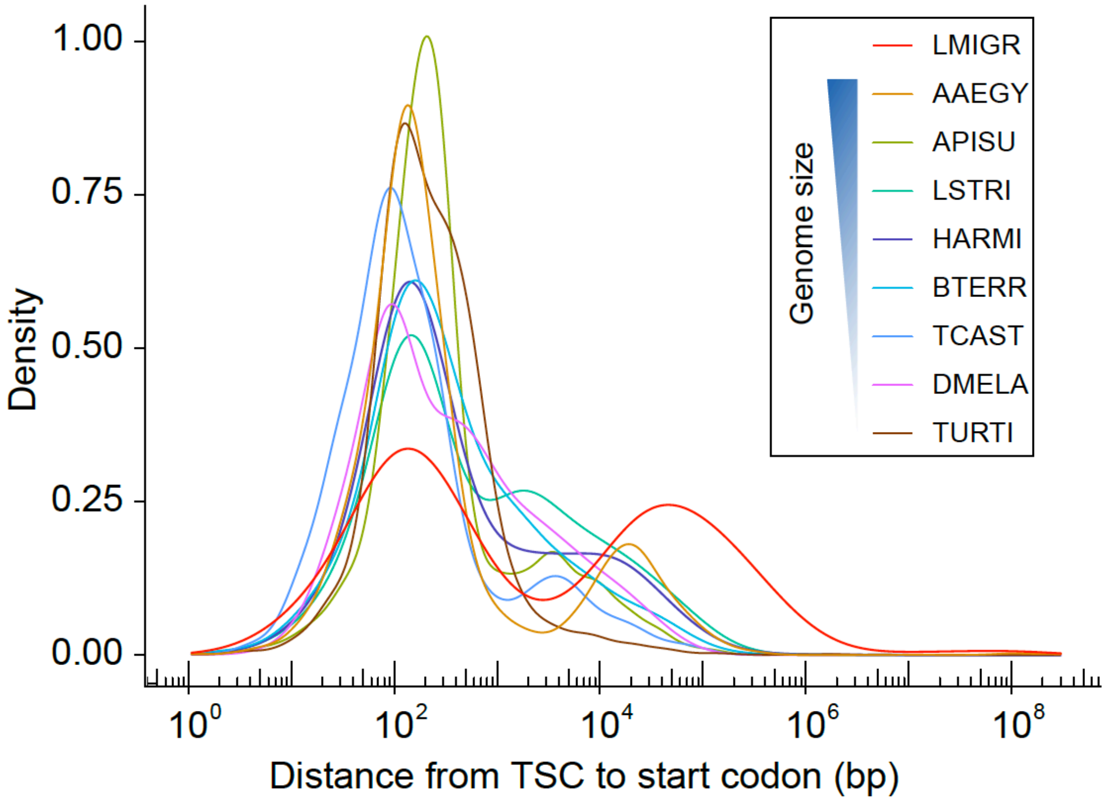

Fig. S27. The density distribution of distances from the annotated start codon of protein-coding genes to the upstream genic core promoters. *Aedes aegypti*, AAEGY; *Acyrthosiphon pisum*, APISU; *Bombus terrestris*, BTERR; *Helicoverpa armigera*, HARMI; *Laodelphax striatellus*, LSTRI; *Tribolium castaneum*, TCAST; *Tetranychus urticae*, TURTI.

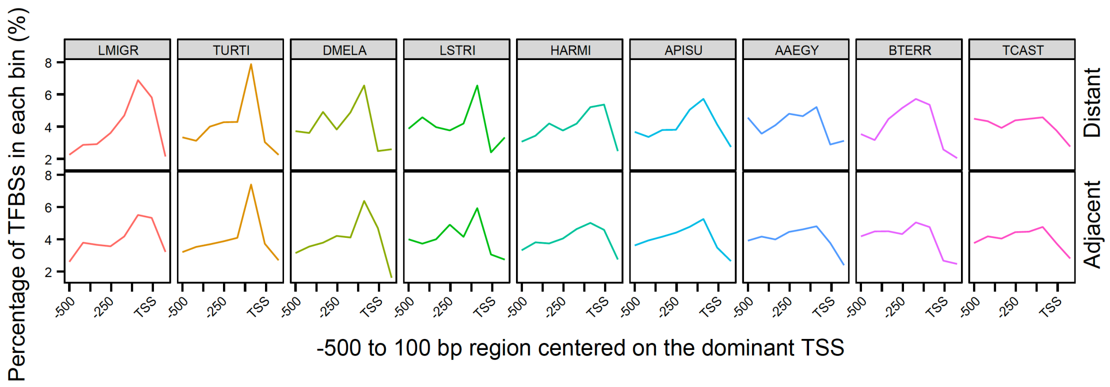

Fig. S28. The abundance distribution of the distances from the TFBSs to the dominant transcription starting site (TSS) in protein-coding genes. The dominant TSSs located less than 125 bp upstream from the start codons were not included in this analysis. *Aedes aegypti*, AAEGY; *Acyrthosiphon pisum*, APISU; *Bombus terrestris*, BTERR; *Helicoverpa armigera*, HARMI; *Laodelphax striatellus*, LSTRI; *Tribolium castaneum*, TCAST; *Tetranychus urticae*, TURTI.

Supplemental Tables

Table S1. Sequencing data generated in this study for locusts.

Library	Number of sequencing reads	Bases (Gb)
Antenna-1	125,699,634	18.85
Brain-1	127,902,546	19.19
Fatbody-1	188,244,664	28.24
Labipalp-1	136,653,642	20.50
Labipalp-2	106,032,404	15.90
Thoracic muscle-1	179,122,214	26.87
Thoracic muscle-2	149,911,360	22.49
Ovary-1	129,924,214	19.49
Pronotum-1	135,537,778	20.33
Pronotum-2	121,662,468	18.25
Testis-1	143,578,290	21.54
Testis-2	85,467,274	12.82
Wing-1	142,203,706	21.33
Wing-2	121,088,596	18.16
Total	1,893,028,790	283.95

Table S2. Overrepresented motifs of TGAG and its variants in the 1-bp-wide TSCs located in the intergenic region. The significance of overrepresented motifs in the 1-bp-wide TSCs located in the intergenic region was tested using the Fisher's exact test, which was applied by the qvalue package in R. "# Observed" and "% Observed" represent the motif number in the 1-bp-wide TSCs and the percentage relative to the total number of the 1-bp-wide TSCs in the intergenic region, respectively. "# Mismatch" represents the substitution number compared to the TGAG motif. The motifs of which the observed number is less than 10 are not shown.

Motifs	# Mismatch	# Observed	% Observed	# (in 5'-UTR)	%	Q-value
TGAG	0	15714	38.56	4	0.11	0.00E+00
AGAG	1	4045	9.93	2	0.06	2.68E-149
GGAG	1	2698	6.62	2	0.06	1.50E-96
CGAG	1	2495	6.12	4	0.11	4.21E-85
TGAA	1	722	1.77	2	0.06	8.38E-23
TGTG	1	443	1.09	4	0.11	3.88E-11
AAAG	2	345	0.85	4	0.11	4.58E-08
CAAG	2	306	0.75	3	0.08	1.16E-07
GAAG	2	210	0.52	2	0.06	1.46E-05
ACAG	2	190	0.47	1	0.03	8.33E-06
ATAG	2	111	0.27	1	0.03	2.72E-03
GCAG	2	44	0.11	1	0.03	3.79E-01
AGGG	2	43	0.11	2	0.06	6.52E-01
AGTG	2	42	0.10	2	0.06	6.52E-01
CCAG	2	42	0.10	1	0.03	3.79E-01
AGAA	2	37	0.09	1	0.03	4.67E-01
CGTG	2	36	0.09	1	0.03	4.67E-01
AGCG	2	25	0.06	1	0.03	7.69E-01

GGCG	2	18	0.04	1	0.03	1.00E+00

Table S3. Over-represented motifs of TGAG and its variants in the non-1-bp-wide TSCs located in the intergenic region. The significance of overrepresented motifs in the non-1-bp-wide TSCs located in the intergenic region was tested using Fisher's exact test, which was applied by the qvalue package in R. "# Observed" and "% Observed" represent the motif number in the non-1-bp-wide TSCs and the percentage relative to the total number of the non-1-bp-wide TSCs in the intergenic region, respectively. "# Mismatch" represents the substitution number compared to the TGAG motif. The motifs of which the observed number is less than 10 are not shown.

Motifs	# Mismatch	# Observed	% Observed	# (in 5'-UTR)	%	Q-value
TGAG	0	1469	9.20	4	0.11	1.07E-124
AGAG	1	642	4.02	2	0.06	1.81E-52
GGAG	1	612	3.83	2	0.06	4.30E-50
CGAG	1	501	3.14	4	0.11	2.11E-37
TGCC	2	115	0.72	21	0.59	5.91E-01
CGAC	2	111	0.70	24	0.67	1.00E+00
TGAA	1	107	0.67	2	0.06	1.32E-06
TGTG	1	87	0.54	4	0.11	9.15E-04
GGAC	2	77	0.48	16	0.45	9.34E-01
GAAG	2	44	0.28	2	0.06	4.42E-02
CGGG	2	41	0.26	5	0.14	4.13E-01
AAAG	2	40	0.25	4	0.11	3.19E-01
ACAG	2	40	0.25	1	0.03	1.70E-02
CAAG	2	35	0.22	3	0.08	3.02E-01
AGGG	2	32	0.20	2	0.06	2.10E-01
GGAT	2	31	0.19	4	0.11	5.69E-01

TCAC	2	29	0.18	5	0.14	8.81E-01
GCAG	2	22	0.14	1	0.03	2.66E-01
CGTG	2	21	0.13	1	0.03	3.19E-01
TTAT	2	20	0.13	4	0.11	1.00E+00
AGTG	2	19	0.12	2	0.06	5.82E-01
GGCG	2	19	0.12	1	0.03	3.19E-01
ATAG	2	17	0.11	1	0.03	3.87E-01
AGAA	2	16	0.10	1	0.03	5.23E-01
CCAG	2	14	0.09	1	0.03	5.23E-01

Table S4. Overrepresented motifs of TGAG and its variants in the 1-bp-wide TSCs located in the intronic region. The significance of overrepresented motifs in the 1-bp-wide TSCs located in the intronic region was tested using Fisher's exact test, which was applied by the qvalue package in R. "# Observed" and "% Observed" represent the motif number in the 1-bp-wide TSCs and the percentage relative to the total number of the 1-bp-wide TSCs in the intronic region, respectively. "# Mismatch" represents the substitution number compared to the TGAG motif. The motifs of which the observed number is less than 10 are not shown.

Motifs	# Mismatch	# Observed	% Observed	# (in 5'-UTR)	%	Q-value
TGAG	0	2039	44.18	4	0.11	0.00E+00
AGAG	1	80	1.73	2	0.06	2.71E-16
CGAG	1	78	1.69	4	0.11	3.77E-14
GGAG	1	59	1.28	2	0.06	9.78E-12
TGAA	1	28	0.61	2	0.06	8.81E-05

Table S5. Over-represented motifs of TGAG and its variants in the non-1-bp-wide TSCs located in the intronic region. The significance of overrepresented motifs in the non-1-bp-wide TSCs located in the intronic region was tested using Fisher's exact test, which was applied by the qvalue package in R. "# Observed" and "% Observed" represent the motif number in the non-1-bp-wide TSCs and the percentage relative to the total number of the non-1-bp-wide TSCs in the intronic region, respectively. "# Mismatch" represents the substitution number compared to the TGAG motif. The motifs of which the observed number is less than 10 are not shown.

Motifs	# Mismatch	# Observed	% Observed	# (in 5'-UTR)	%	Q-value
TGAG	0	30	1.42	4	0.11	3.37E-08
TGGC	2	24	1.14	39	1.09	1.00E+00
CGAC	2	22	1.04	24	0.67	5.01E-01
TGCC	2	22	1.04	21	0.59	3.83E-01
AGAG	1	17	0.81	2	0.06	6.58E-05
TAAC	2	16	0.76	14	0.39	4.75E-01
GGAC	2	14	0.66	16	0.45	7.88E-01
GGAG	1	13	0.62	2	0.06	1.47E-03
CGAT	2	12	0.57	16	0.45	1.00E+00

Table S6. Over-represented motifs of TGAG and its variants in the 1-bp-wide TSCs located in the coding sequence (CDS) region. The significance of overrepresented motifs in the 1-bp-wide TSCs located in the CDS region was tested using Fisher's exact test, which was applied by the qvalue package in R. "# Observed" and "% Observed" represent the motif number in the 1-bp-wide TSCs and the percentage relative to the total number of the 1-bp-wide TSCs in the CDS region, respectively. "# Mismatch" represents the substitution number compared to the TGAG motif. The motifs of which the observed number is less than 10 are not shown.

Motifs	# Mismatch	# Observed	% Observed	# (in 5'-UTR)	%	Q-value
TGAG	0	403	18.75	4	0.11	3.64E-173
CGAG	1	71	3.30	4	0.11	1.43E-24
GGAG	1	56	2.61	2	0.06	1.00E-20
AGAG	1	38	1.77	2	0.06	2.02E-13
CGAC	2	25	1.16	24	0.67	1.27E-01
TGAA	1	14	0.65	2	0.06	3.50E-04
ACAG	2	12	0.56	1	0.03	3.65E-04
GGAC	2	12	0.56	16	0.45	8.26E-01

Table S7. Overrepresented motifs of TGAG and its variants in the non-1-bp-wide TSCs located in the coding sequence (CDS) region. The significance of overrepresented motifs in the non-1-bp-wide TSCs located in the CDS region was tested using Fisher's exact test, which was applied by the qvalue package in R. "# Observed" and "% Observed" represent the motif number in the non-1-bp-wide TSCs and the percentage relative to the total number of the non-1-bp-wide TSCs in the CDS region, respectively. "# Mismatch" represents the substitution number compared to the TGAG motif. The motifs of which the observed number is less than 10 are not shown.

Motifs	# Mismatch	# Observed	% Observed	# (in 5'-UTR)	%	Q-value
TGAG	0	53	2.17	4	0.11	3.12E-15
CGAC	2	30	1.23	24	0.67	1.34E-01
GGAG	1	29	1.19	2	0.06	1.57E-08
AGAC	2	28	1.15	36	1.01	8.15E-01
TGCC	2	24	0.98	21	0.59	2.75E-01
CGAG	1	21	0.86	4	0.11	1.39E-04
GGAC	2	21	0.86	16	0.45	1.99E-01
TGCT	2	21	0.86	26	0.73	8.17E-01
AGAG	1	13	0.53	2	0.06	2.91E-03

Table S8. Consensus sequences of *Drosophila* core promoter elements.

Motif	Location	Consensus
TATA-box	Upstream T at -32 to -28	TATAWR
Inr	-2 to +4	TCA+1GTY
TCT	-2 to +6	YYC+1TTTYY
MTE	+18 to +22	CGANC
	+27 to +29	CGG
DPE	+28 to +32	RGWYV

Inr, initiator; TCT, polypyrimidine initiator; MTE, motif ten element; DPE, downstream core promoter element. The consensus sequences of *Drosophila* core promoter elements were retrieved from a recent review. The mismatch in the A+1 of Inr motif and in the C+1 of TCT motif was not allowed in the analysis.

Table S9. Sequencing data generated in this study for the arthropod species.

Library	Number of sequencing reads	Bases (Gb)
AAEGY-1	83,434,138	12.52
AAEGY-2	173,362,120	26.00
APISU-1	65,365,188	9.80
BTERR-1	64,538,324	9.68
BTERR-2	117,397,290	17.61
HARMI-1	75,429,380	11.31
HARMI-2	115,618,132	17.34
LSTRI-1	54,043,278	8.11
LSTRI-2	59,936,498	8.99
TCAST-1	76,693,726	11.50
TCAST-2	145,136,828	21.77
TURTI-1	82,970,286	12.45
TURTI-2	122,620,388	18.39

Aedes aegypti, AAEGY; *Acyrthosiphon pisum*, APISU; *Bombus terrestris*, BTERR; *Helicoverpa armigera*, HARMI; *Laodelphax striatellus*, LSTRI; *Tribolium castaneum*, TCAST; *Tetranychus urticae*, TURTI.

Table S10. The identified TSCs in the arthropod species.

Species	TSCs	Genic TSCs
Aedes aegypti	15,915	7,367
Acyrthosiphon pisum	22,361	10,130
Bombus terrestris	21,872	7,900
Helicoverpa armigera	17,636	10,850
Laodelphax striatellus	11,107	8,197
Tribolium castaneum	16,784	12,384
Tetranychus urticae	12,524	9,893
